# Impact of Endemic Infections on HIV Susceptibility in Sub-Saharan Africa

**DOI:** 10.1186/s40794-019-0097-5

**Published:** 2019-11-29

**Authors:** Sergey Yegorov, Vineet Joag, Ronald M. Galiwango, Sara V. Good, Brenda Okech, Rupert Kaul

**Affiliations:** 1 Departments of Immunology and Medicine, University of Toronto, Toronto, Canada; 2 Department of Pedagogical Mathematics and Natural Science, Faculty of Education and Humanities, Suleyman Demirel University, Almaty, Kazakhstan; 3 Department of Microbiology and Immunology, University of Minnesota, Minneapolis, MN USA; 4 Genetics & Genome Biology, The Hospital for Sick Children, Peter Gilgan Centre for Research and Learning, Toronto, ON Canada; 5 Community Health Sciences, University of Manitoba, Winnipeg, MB Canada; 6 UVRI-IAVI HIV Vaccine Program, Entebbe, Uganda; 7 Department of Medicine, University Health Network, Toronto, Canada

**Keywords:** HIV susceptibility, HIV risk factors, Sub-Saharan Africa, parasitic infections, malaria, helminthiases, lymphatic filariasis, schistosomiasis

## Abstract

Human immunodeficiency virus (HIV) remains a leading cause of global morbidity with the highest burden in Sub-Saharan Africa (SSA). For reasons that are incompletely understood, the likelihood of HIV transmission is several fold higher in SSA than in higher income countries, and most of these infections are acquired by young women. Residents of SSA are also exposed to a variety of endemic infections, such as malaria and various helminthiases that could influence mucosal and systemic immunology. Since these immune parameters are important determinants of HIV acquisition and progression, this review explores the possible effects of endemic infections on HIV susceptibility and summarizes current knowledge of the epidemiology and underlying immunological mechanisms by which endemic infections could impact HIV acquisition. A better understanding of the interaction between endemic infections and HIV may enhance HIV prevention programs in SSA.

## Introduction and overview

Despite the ability of antiretroviral therapy (ART) to reduce HIV infection at an individual level, its impact on HIV transmission at a population level has been limited. In 2017 the global number of new HIV infections remained at 1.8 million, which lags far behind the pace needed to reach the UNAIDS Fast-Track Target of fewer than 500,000 new infections per year by 2020 [1]. Notably, over 36 million people are currently living with HIV [1], underscoring the importance of developing new and affordable HIV prevention strategies with the potential for broad scale up.

Individuals from lower-income countries, particularly those in Sub-Saharan Africa (SSA), exhibit a nearly 4-fold elevated risk of male-to-female HIV transmission per sexual contact compared to higher income countries (0.3% versus 0.08%) [2]. The reason for this difference is not clear, but likely relates to numerous factors that may include circulating virus characteristics, socio-behavioral patterns and environmental factors. Additional parameters that have been linked to HIV transmission risk and may be more common in individuals from SSA include the use of injectable hormonal contraceptives [3], alterations in the vaginal microbiome including bacterial vaginosis (BV) [4], and a higher population prevalence of sexually transmitted infections such as herpes simplex type 2 (HSV-2) [5, 6].

Even within SSA there is tremendous regional heterogeneity in HIV transmission. For example, in East African countries the incidence of HIV is increased almost ten-fold near Lake Victoria [5]. A possible reason for this regional heterogeneity may be the biological impact on HIV susceptibility of common non-genital infections, such as malaria, helminthiases, tuberculosis and others [6]. The prevalence of these infections can vary considerably within a region, and they have been previously demonstrated to increase the blood HIV viral load in infected, ART-naïve people, rendering it more likely that co-infected individuals will transmit HIV to their partners [7, 8].

To date, the effect of such endemic diseases on HIV transmission has been explored mainly in the context of co-infection in HIV-infected individuals and predominantly by looking at the impact of co-infections and their treatment on blood HIV load [7–9], because viral load is the key determinant of HIV transmission probability from an HIV-infected person to their HIV-uninfected sexual partner [10]. However, much less is understood about the potential impact of endemic infections on an HIV-uninfected individual’s HIV susceptibility, despite accumulating epidemiological and biological evidence for such effects. Given that endemic pathogens in SSA infect many HIV at-risk individuals, a clear understanding of their potential influence on HIV susceptibility may aid in the development of better disease prevention strategies, as is envisioned in recent proposals for integrated disease control in high disease burden regions [11–14].

This review focuses on the effects of non-STI endemic pathogens (Table 1), many of which cause chronic and asymptomatic infection, on HIV susceptibility in SSA. First, we review the biological characteristics that define HIV susceptibility, specifically focusing on the sexual acquisition of HIV in the genital tract and rectum. Secondly, we discuss the evidence available to date on the relationship between select endemic infections and HIV susceptibility using malaria and helminth infections as examples. Finally, we discuss whether and how treatment and prevention of endemic infections could help reduce HIV acquisition and ultimately alleviate HIV burden in SSA.

**Table 1:** Endemic infections that may enhance HIV susceptibility in Sub-Saharan Africa

Infection	Main causative agent (s) in SSA	Primary tissue site(s) involved
Malaria	*Plasmodium* spp.- mainly *P. falciparum*	Blood, liver
Soil transmitted helminthiases	*Ascaris, Trichuris*, hookworms (e.g. *Necator americanus*)	Gut lumen
Lymphatic filariasis	*Wuchereria bancrofti*	Lymphatic system, blood
Schistosomiasis	*Schistosoma* spp.	
Intestinal schistosomiasis	*S. mansoni*	Blood vessels surrounding gut, gut mucosa
Genitourinary schistosomiasis	*S. haematobium*	Blood vessels surrounding genitourinary tract, genitourinary mucosa

## Biological characteristics that define HIV susceptibility

### Mucosal HIV acquisition: sites of exposure and immunological correlates of risk

HIV is most commonly (85%) acquired via contact with virus-containing bodily fluids through unprotected sex [15], and heterosexual sex accounts for the majority of sexual HIV transmission in SSA [15–21]. In many SSA countries young women are at especially high risk of HIV acquisition, with an incidence 3-4 times higher than their male peers [22–24]. The high incidence of HIV in women from SSA is difficult to reconcile with the relatively low per-contact probability of male-to-female HIV transmission, which is estimated to range from 1/250 to 1/2500 [2]. While this low transmission likelihood is attributable to several effective defense mechanisms in the female genital tract [25], community-wide factors that modify HIV susceptibility can still result in a high population HIV incidence. These modifying factors are discussed below.

### HIV acquisition in the female genital tract

The female genital tract (FGT) is often sub-divided into the lower genital tract (the vagina and ectocervix), which is covered by a squamous epithelium, and the upper genital tract, which is covered with columnar epithelium (the endocervix, uterus, fallopian tubes and ovaries). The lower genital tract and the “transition zone” between the lower and upper genital mucosa have traditionally been thought of as the main sites of HIV acquisition, although studies in the macaque model suggest that both the entire upper and lower genital mucosa may be susceptible to HIV [26–28].

In primate models [15, 29, 30], virus crosses the genital epithelium within several hours of exposure to an infectious inoculum, either through breaches in the mucosa or by diffusing across an intact stratified squamous epithelium, and can be found in proximity to target cells in the lamina propria [31], although in theory direct dissemination to the bloodstream is possible if the viral challenge dose is high. Next, a “founder” population of infected cells, composed mainly of CD4+ T cells, expands within the mucosa for approximately the first week after challenge, followed by dissemination to tissue draining lymph nodes; once here, host infection has been irreversibly established and the virus rapidly spreads throughout the body, replicating at particularly high levels within gut-associated lymph tissues [28, 32–34]. Notably, some studies suggest an even rapider scenario of viral dissemination to distal organs occurring within a few hours after exposure [35].

Thus, the first week after exposure, also termed the “window of HIV vulnerability”, is likely to be critical for the success of preventive strategies that could be deployed to stop viral infection and spread [36]. Some of these strategies, such as ART-based pre-exposure and post-exposure prophylaxis, which limit local virus replication, have already shown efficacy in humans [37, 38]. Other approaches, such as mucosal induction of antiviral pathways [39], blockade of target cell migration [40–42], induction of broadly neutralizing antibodies [43] and stimulation of T cell-mediated responses [44] so far have shown promising results and could potentially exert a strong impact on the HIV window of vulnerability. Antiviral pathway functionality and target cell migration mechanisms can be modified by various biological factors, such as co-infections, and are therefore important contributors to changes in HIV susceptibility.

### HIV acquisition in the male genital tract

Among heterosexual men, the penis is the key organ at which virtually all HIV infections are acquired. Throughout the penile tissue, abundant macrophages and Langerhans cells comprise the main antigen presenting cells and together with plasma and T cells mediate adaptive immune responses [45–52]. In heterosexual men, circumcision reduces HIV incidence by 50-60% [22–24], suggesting that the foreskin is an important site of HIV acquisition, although other penile tissues such as the urethra may also play a role in HIV acquisition [53, 54]. Since the foreskin tissues are not mucosal in the traditional sense because they lack mucus-secreting capacity and are keratinized, it is possible that the effects of endemic mucosal infections on penile HIV susceptibility might be quite different to vaginal and rectal tissues. In addition to the anatomical differences, differences in CD4+ T cell trafficking to the foreskin versus the female genital tissues may also explain differences in HIV susceptibility of men versus women [48, 55].

### HIV acquisition in the rectum

Both men and women may acquire HIV across the rectal mucosa during receptive anal intercourse (AI). Unprotected AI is common in SSA [56] and is a high risk factor for HIV infection [57]. The rectal mucosa is a gateway to a large pool of HIV target cells, such as CCR5+ macrophages and CD4 T cells, with high proportions of Th17 cells [58, 59]. Notably, recent research indicates that compared to people who had never engaged in AI, the rectal mucosa of individuals engaging in unprotected AI also exhibits a phenotype indicative of elevated inflammation and mucosal injury [58]. This is important, since multiple endemic infections in SSA involve the gut and could therefore enhance HIV susceptibility via this route.

### Cellular correlates of HIV susceptibility

At the cellular level, some of the major determinants of susceptibility to HIV infection include the surface expression of the primary HIV receptor (CD4), expression of the co-receptors CCR5 or CXCR4, the production of various innate antiviral factors, and the physical localization of the cell. HIV transmission via mucosal routes is almost always mediated by CCR5-tropic rather than CXCR4-tropic viral variants, despite the frequent presence of both variants in the genital secretions of an infected person and the expression of both CXCR4 and CCR5 on mucosal CD4+ T cells from an uninfected sex partner [60–62]. The reason for such strong selection bias in favour of CCR5-tropism is likely multifactorial [60, 61], and is beyond the scope of this review. The activation state of mucosal CD4 T cells is a critical determinant of HIV susceptibility, with activated effector and memory CD4 T cells constituting preferential targets [59, 63, 64], and is often assessed experimentally through the surface expression of CD38, HLA-DR, Ki-67 and/or CD69 [65, 66].

In addition to their activation status and co-receptor expression, CD4 T helper (Th) cells can be classified into multiple subsets based on their immune functions and the expression of specific transcription factors and surface receptors, and there are clear subset differences in susceptibility to HIV [59, 67]. The main mucosal target for HIV infection is Th17 cells, which abundantly express HIV receptors/co-receptors and integrin α4β7, but lack CCR5 ligand expression and exhibit reduced intrinsic capacity to inhibit HIV replication [68]. Th17 cells are primarily involved in host mucosal defense against bacteria and yeast, and are defined based on expression of the transcription factors retinoic acid related orphan receptor (ROR)-γt/RORC and ROR-α, the surface expression of the chemokine receptor CCR6, and the production of IL-17 [69, 70]. In a macaque SIV infection model up to 85% of early virus-infected cells in the genital tract are CCR6+ [71], and in humans cervical IL-17+ cells are dramatically depleted very early in the course of human HIV infection [72], with preferential HIV infection of genital CCR6+ cells *in vitro* [73].

### T cell integrins, the common mucosal immune system, and HIV susceptibility

Another correlate of cellular HIV susceptibility is the expression of integrin heterodimers, particularly the mucosal homing integrin α4β7, which both homes cells to mucosal sites through binding to MAdCAM [74] and can also facilitate binding of HIV to target cells [75]. Integrins are transmembrane glycoproteins that enable cell adhesion to the extracellular matrix and direct cell trafficking and retention in various anatomical sites [76]. Together with their ligands, integrins play a key role in the “common mucosal immune system” [77–79], which facilitates linkage and cross-talk between the immune cells of the gastrointestinal, respiratory and urogenital mucosae. As a result, an immune response generated at one mucosal site may in some cases induce a response in an anatomically distinct mucosal site via tissue homing through common mucosal pathways. For instance, oral immunization can generate an antibody response in the small intestine [77], and nasal immunization can induce host immune responses in the respiratory and reproductive tracts [77, 80–82]. Furthermore, systemic vaccination can induce high levels of mucosal homing T cells in the blood, with subsequent protection seen against genital virus challenge [83]. The three integrins α4β7 (CD49d/β7), α4β1 (CD49d/CD29) and αEβ7 (CD103/β7) appear to be especially important for mucosal T cell localization [84], which has implications for HIV pathogenesis, and since these parameters are also influenced by parasitic infections [85, 86], this could have important implications for the effect of endemic infections on HIV transmission.

### Antiviral defense mechanisms, mucosal microbiota and HIV susceptibility

Intact mucosal surfaces present multiple lines of defense against viral invasion, such as an intact cervicovaginal epithelium, low pH mucus containing immunoglobulins, antimicrobial peptides and tissue resident immune cells that drive innate and adaptive antiviral responses [87]. The cervicovaginal mucus, for example, presents a physical obstruction for pathogens like HIV, as it can trap the virus at acidic pH [88]. Acidification of the cervicovaginal milieu is caused by lactic acid production by commensal lactobacilli and is thought to play an important role in HIV susceptibility [88].

Genital microbiota influence both genital immunology and HIV susceptibility. Bacterial vaginosis (BV), for example, is a commonly encountered alteration of the vaginal microbiome causing vaginal discharge. BV is associated with an augmented risk of acquiring sexually transmitted infections (STIs) including HIV [4, 89]. In line with this, research from South and East Africa shows that the genital abundance of dysbiosis-associated bacteria is associated with elevated odds of HIV acquisition [90, 91], while HIV-uninfected women with lactobacillus-predominant microflora are less likely to acquire HIV [91, 92] and have an improved efficacy of HIV pre-exposure prophylaxis [93].

Systemically and in mucosae multiple innate antiviral defense mechanisms are effectively regulated by the interferon (IFN) system [94, 95]. Resistance to type I IFNs is recognized as a key characteristic of some early-transmitted HIV strains [96], while both IFN-II and III are recognized for their direct antiviral activity and ability to modulate antiviral immune responses [97–99]. Since parasitic infections can alter both mucosal microbial environment [100–105] and innate antiviral signalling [106–108], this could have implications for anti-HIV defense mechanisms.

### Inflammation and HIV susceptibility

Inflammation is a complex immunological response to tissue damage and/or pathogen invasion, which ultimately aims to restore tissue integrity and eliminate the infection. A typical proinflammatory response involves cytokine production by epithelial, innate and adaptive immune cells, which leads to extravasation and further activation of immune cells at the tissue site. While an effective antiviral response involves immune activation, as seen for example during the induction of IFN-I signalling- where despite an increase in the number of target cells HIV infection is reduced [39, 109], chronic inflammation is thought to enhance HIV acquisition risk through various mechanisms. In the genital mucosa, persistent inflammation may disrupt cellular junctions and thus increase epithelial barrier permeability, which could facilitate HIV access to mucosal target cells [110]. At the same time, persistently elevated numbers of activated CD4 T cells at HIV exposure sites, as seen, for example, in sexually transmitted infections, would supply more cell targets for the virus [59]. Importantly, chronic inflammation also suppresses antiviral defenses and dysregulates interferon signaling [94]. In keeping with the detrimental effects of chronic inflammation on HIV immunity, pre-existing genital [111, 112] and systemic [113, 114] immune activation has been associated with subsequent HIV acquisition. On the other hand, HIV-exposed seronegative individuals (HESN), who may be less susceptible to HIV acquisition after sexual exposure, appear to have lowered systemic and mucosal immune activation compared to HIV-uninfected controls [52, 115–118]. Although some other studies have found that HESN have elevated levels of circulating HLA-DR+ T cells and CCR5+ CD4+ T cells [119] and increased CCR5 expression in cervical biopsies [120], as well as a high prevalence of genital co-infections acquired due to shared sexual risk factors.

#### Soluble immune mediators as biomarkers of HIV susceptibility

The impact of a pathogen on the genital or blood levels of soluble immune mediators can shed light on the likely influence of this pathogen on HIV susceptibility in human cohorts. However, it is important to remember that co-infections and behavioural factors may have differential effects on the immune parameters in the systemic and mucosal compartments [112, 121–123], and it is the immune milieu at the site of HIV exposure that is most relevant to HIV susceptibility [124].

The detection and/or level of several systemic and mucosal cytokines have been associated with HIV susceptibility in both human and macaque studies [112–114, 125, 126]. For example, HIV acquisition in South African women was associated with increased levels of vaginal genital macrophage inflammatory protein (MIP)-1α, MIP-1β and IFN-γ-induced protein (IP)-10 [112], as well as increased mucosa-to-blood ratios of IP-10, MIP-1β, IL-8, granulocyte-macrophage colony-stimulating factor (GM-CSF) and monocyte chemoattractant protein (MCP)-1 [125]. Another study found that blood levels of TNF, IL-2, IL-7 and IL-12 were increased in women who subsequently acquired HIV, compared with their female peers who remained HIV-uninfected [113]. Interestingly, a study of HIV-discordant couples from six different African countries reported an association of elevated circulating IL-10 and IP-10 with HIV seroconversion [114], although in South African women systemic IP-10 was inversely associated with HIV risk [125]. The reasons for these discrepancies are unclear, but this emphasizes the importance of studying immune factors at the actual mucosal site of HIV exposure rather than in blood, where associations may be confounded by genetic or environmental factors. Lastly, in a model of rectal simian HIV infection, systemic IL-8, RANTES (regulated on activation, normal T cell expressed and secreted) and eotaxin concentrations were associated with resistance to viral infection, while detectable blood IL-6 was associated with elevated susceptibility [126].

Despite some study-to-study variability, overall these data suggest that increased levels of mucosal proinflammatory cytokines are associated with elevated HIV susceptibility, in keeping with the association of these cytokines with mucosal epithelial barrier perturbations and influx of HIV target cells [110]. On the other hand, the relationship of systemic immune mediators with HIV susceptibility is distinct and less consistent than that of mucosal cytokines.

## The relationship between endemic infections and HIV susceptibility

In this part of the review we will discuss the epidemiological and experimental data available on the relationship between endemic infections and HIV susceptibility using two fundamentally different but frequently ecologically overlapping examples of endemic infections: i) a protozoan infection (malaria) and ii) infections by multicellular parasites (helminths).

## Malaria and HIV susceptibility

Malaria is caused by protozoan parasites of genus *Plasmodium* transmitted via a bite of Anopheles mosquito. Most malaria-associated morbidity in SSA is due to *Plasmodium falciparum,* the most prevalent malarial parasite in the region [127]. *Plasmodium* infects red blood cells and causes a febrile response in the infected individual. Without timely treatment, the disease can result in severe manifestations and even death. Residents of regions with stable malaria transmission rates over time become partially immune, are able to maintain low levels of infection and exhibit “asymptomatic” or “sub-clinical” malaria [128]. According to the WHO, in 2016 more than 190 million cases of malaria caused over 400,000 deaths in Africa [129]. Since the infection is more likely to cause severe manifestations in children than in adults, true malaria cases in adults are under-reported resulting in inaccurate estimations of disease burden [127]. Moreover, due to the difficulties encountered with maintaining high standards of diagnostic testing, malaria is frequently misdiagnosed resulting in high rates of false positivity in the absence of true infection, which can present barriers for clinical trials [130–132].

### Impact of malaria on HIV susceptibility: epidemiological evidence

With the exception of South Africa where malaria transmission is infrequent, there is considerable ecological overlap between malaria and HIV transmission in SSA [133]. A cross-sectional study of 907 Tanzanian adults with and without HIV or malaria, found a significant association between malaria and HIV infection in a region with HIV and *P. falciparum* prevalence of 7.9% and 12.3%, respectively [134], providing evidence for an interaction between malaria and HIV transmission in a population with mixed HIV status. Аnother study based on the HIV and *P. falciparum* distribution in East Africa found that the residents of regions with high *P. falciparum* rates (Pf parasite rate (PfPR) >0.42) have a 2.44 fold risk of being HIV-infected compared to individuals living in low *P. falciparum* transmission settings (PfPR<0.01) after adjusting for social and biological risk factors associated with both infections [135]. Notably, this effect of malaria was not gender-specific (adjusted p<0.001 in both men and women), suggesting a biological mechanism that is dominated by systemic effects of malaria on HIV susceptibility. Further, the effect seen in the study was similar in magnitude to the association seen for genital ulcers in the same study [135]. Interestingly, the same group reported a lack of association between malaria and HIV in West African countries, attributing this discrepancy to region-specific HIV dynamics and the lower HIV prevalence in West Africa (≤5.0 %) compared to East Africa (≤10%) [136] and due to differences in the replicative capacity and infectiousness of the HIV subtypes dominant in these regions [137], while additional important factors could be the difference in circumcision rates between East and West Africa as well as other behavioural and environmental differences between the regions.

The mechanism for malaria-HIV interaction has been assumed to be the effect of malaria on HIV viral load and infectiousness in a co-infected person. A study from Malawi demonstrated that in co-infected individuals febrile malaria caused a ten-fold increase in HIV load [138], which translates into a roughly 2.5-fold increase in HIV transmission probability [10]. Applying these data and mathematical modelling to the western Kenyan community of Kisumu, Abu-Raddad and colleagues estimated that over 8500 HIV infections occurred in a Kenyan community of ~200,000 people because of malaria-HIV viral load interaction over a period of a decade [139], making the population attributable fraction of HIV cases attributable to malaria roughly 20%.

### Impact of malaria on HIV susceptibility: potential immunological mechanisms

How malaria, a predominantly systemic condition, could influence mucosal HIV transmission is not completely clear, although several lines of evidence suggest that elevated immune activation is a major underlying cause. Thus, plasmodial antigens [140] and parasitized red blood cells [141] induce production of pro-inflammatory cytokines, such as tumor necrosis factor (TNF), in blood mononuclear cells leading to increased T cell activation and elevated HIV replication *in vitro* [140]. In HIV-infected individuals malaria-induced immune activation drives elevation of blood viral load [138, 142], substantially enhancing viral transmission to HIV-uninfected partners [139].

While the effects of malaria on HIV susceptibility have not been assessed in HIV-negative people, there is compelling evidence from a murine model that malaria-associated systemic immune activation also translates into inflammation at mucosal interfaces. In particular, a study by Chege *et al.* found that malaria-infected mice had a more than four-fold increase in the number of activated/CCR5+ CD4 T cells in the genital tract, as well as substantial increases in the blood and gut mucosa [86]. Moreover, infected animals exhibited increased frequencies of circulating α4β7+ CD4 T cells, suggesting that malaria-induced systemic inflammation was enhancing the mucosal homing of activated immune cells [86].

There is also evidence for malaria’s direct pathogenic effects in mucosa. For example, *P. falciparum*-infected individuals show signs of gut epithelial barrier damage leading to increased gastrointestinal permeability [143] and elevated blood levels of lipopolysaccharide (LPS) [144]. These effects appear to be mediated through cytoadherence of infected red blood cells in the gut microvasculature [145], followed by mast cell invasion of intestinal villi and subsequent histamine release affecting intercellular tight junctions [146, 147]. The evidence of intestinal immune perturbations suggests that malaria may result in the induction of shared mucosal pathways, triggering activated immune cells trafficking to other mucosal sites, such as the genital tract. Cumulatively, the data from mouse models and human populations point to a plausible mechanism for malaria-induced elevated HIV susceptibility, which could underpin the malaria-associated HIV infections seen in East African populations.

## Helminth infections and HIV susceptibility

**Figure 1:**
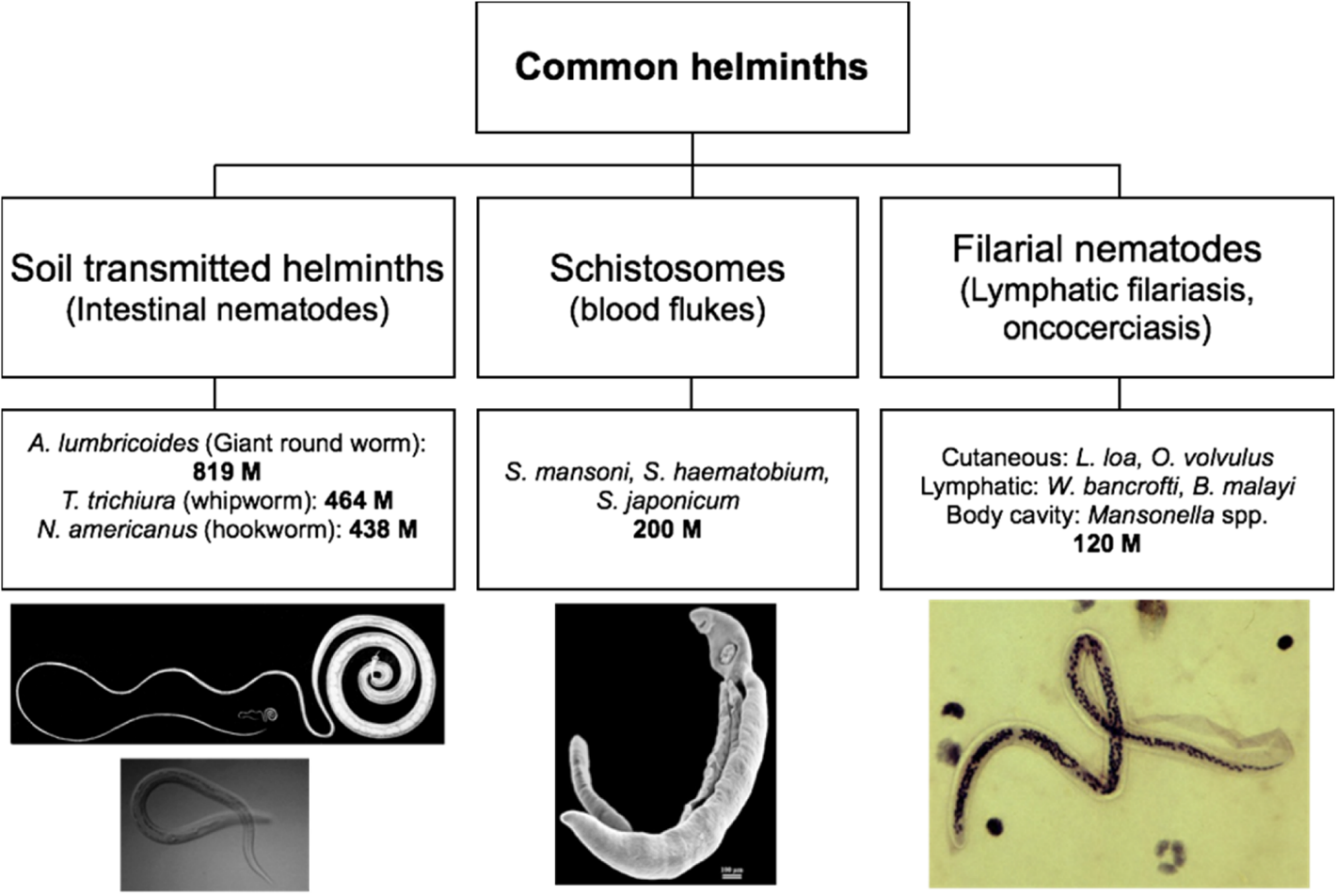
The most common groups of helminths globally. Numbers (in millions, M) refer to the approximate global burden of disease caused by each helminth group. Based on material from http://www.thiswormyworld.org/ and images adapted from Wikimedia. *A. lumbricoides: Ascaris lumbricoides, T. trichiura: Trichuris trichiura, N. americanus: Necator americanus, S. mansoni: Schistosoma mansoni, S. haematobium: Schistosoma haematobium, S. japonicum: Schistosoma japonicum, L. loa: Loa loa, O. volvulus: Onchocerca volvulus, W. bancrofti: Wuchereria bancrofti, B. malayi: Brugia malayi*

Helminths are multicellular organisms that inhabit different anatomical sites in the human host, have complex life cycles. Human infection with worms typically occurs upon contact with contaminated food, water or soil. If left untreated, many helminths cause chronic infection that lasts for many years and results in delayed-onset pathology [148]. Most human morbidity in SSA is associated with three major helminth groups: soil transmitted helminths (STH), schistosomes and filarial nematodes (Fig. 1) [148], all of which are also classified as neglected tropical diseases [148].

In the early 1990s it was proposed that helminthiases could contribute to elevated immune activation and increased HIV susceptibility in African communities [149, 150]. Subsequently, a study compared circulating T cell profiles of Ethiopian immigrants in Israel and found that the recent immigrants, who were heavily infected with helminths, exhibited elevated levels of activated CD4 and CD8 T cells compared to the immigrants who had lived in Israel for several years and were presumably free of helminths [151, 152]. Notably, in these studies the bulk of the recent immigrants were positive for *Schistosoma mansoni* (40.8-51%), followed by soil-transmitted hookworm *Necator americanus* (27.9-49%) and roundworm *Ascaris lumbricoide*s (19.3-35.3%) [151, 152].

In support of these early studies, more recent research identified significant associations between blood T cell activation and soil transmitted helminths *Trichuris* and *Ascaris* [153] as well as evidence for elevated immune activation due to *Wuchereria bancrofti* infection [154]*.* Microbial translocation in the gut appears responsible for the immune activation linked to intestinal helminth infections [155–158]. Interestingly, helminthiasis treatment was associated with reversal of some immunologic parameters such as circulating dendritic cells in participants who cleared hookworm infection [156], but only moderate changes were observed in participants treated for other helminths [153]. Although the area of helminth-HIV interaction remains controversial, the most compelling evidence for the association of helminths and HIV infection comes from several studies of lymphatic filariasis and schistosomiasis (see below).

### Immune response to helminths

Helminth infections are associated with both proinflammatory mucosal immune activation in response to the localized tissue damage due to parasite invasion and strong systemic immune suppression in part mediated by the worms’ own signaling molecules that mimic those found in the host's immunoregulatory apparatus [159].

The two discrete components of the systemic immune system induced by helminths are Th2 and immune regulatory responses, which evolve over time and dominate other effector responses. A Th2 response is marked by elevations in IL-4, IL-5, IL-13, while the hallmark cytokines of the regulatory response are IL-10 and TGFβ [160]. The helminth-induced systemic type 2 and regulatory responses are thought to help helminths escape the host’s proinflammatory responses and reduce tissue damage in chronic infection. For example, the severity of *S. mansoni*-induced granulomatous inflammation is correlated with levels of TNF, soluble TNF receptors and IFNγ, the effects of which are counterbalanced by IL-10 and type 2 cytokines and can lead to severe organ damage in individuals with low Th2 and regulatory responses [161, 162]. The multidimensionality of anti-helminth immune responses is thought to impact immunity to bystander pathogens, such as HIV, and vaccine responses in helminth-infected individuals [163].

Interestingly, the helminth-induced Th2-Treg bias is reminiscent of that observed in chronic HIV infection, where it is thought to favour chronic viral replication [164]; a similar profile is also seen in the genital mucosa of HIV+ women, who exhibit elevated levels of genital IL-4, IL-5 and IL-10 but low levels of IL-2 compared to HIV-uninfected women [165]. Therefore, among the earliest proposed mechanisms to explain helminth effects on HIV infection was helminth-driven shift of Th cell populations to be more Th2-like. It was initially postulated that the Th2 shift on the one hand yields highly HIV-susceptible Th2 cells, and on the other hand suppresses the antiviral Th1 immunity [166]. With advances in cell phenotyping it became clear that Th2 cells are mainly susceptible to CXCR4-tropic HIV [67], rather than the CCR5-tropic strains that are largely responsible for sexual transmission, suggesting that a host Th2 immune bias is unlikely to play a major role in enhanced genital HIV susceptibility; however, helminths induce robust tissue Th17 responses (as described below) [167, 168], providing a more plausible mechanism for helminth-enhanced HIV susceptibility.

### Helminth effects on the host antiviral defense mechanisms and microbiota

Several lines of evidence indicate that parasitic worms may exert profound effects on systemic and mucosal antiviral defenses. Depending on the stage of helminth infection, these effects can promote or suppress the host’s antiviral defense mechanisms. For example, studies in murine models of acute *S. mansoni* and hookworm *Heligmosmoides polygyrus* infection have reported protective effects of these intestinal helminths on antiviral immunity in the lungs [169]. In the case of *S. mansoni* infection this effect was seen in the context of Katayama fever, the highly proinflammatory Th1/Th17 environment that is induced at 10-12 weeks post-helminth infection, and reduced infection by pneumonia and influenza viruses via TNF-mediated mechanisms [170]. In the instance of hookworm infection, the antiviral effects were seen in animals challenged with respiratory syncitial virus 10 days after the helminth infection and were mediated through IFN-I production in both the gut and lung mucosae [169].

In contrast to acute helminth infections, chronic helminthiases appear to dampen host antiviral responses, both via host Treg-Th2 signaling and through the immune-modulatory molecules secreted by the parasites [171]. In line with this, a study of CD4 T cell transcriptomic responses in a murine model of *S. japonicum* infection demonstrated that a significant proportion of schistosome-downregulated host genes belonged to the interferon-inducible gene cluster [172]. The helminth-triggered interferon pathway down-regulation appears to be mediated by the Th2 cytokine IL-4 [173]. In keeping with the latter, infection with intestinal helminth *Trichinella spiralis* diminished immunity to norovirus through IL-4 signaling and STAT6-dependent alternative activation of macrophages with subsequent inhibition of antiviral Th1 function [174].

The role of the microbiome in helminth infection-mediated elevated HIV susceptibility deserves attention in the light of several recently published studies. For example, a study of *S. haematobium*-infected children reported an association of genitourinary schistosomiasis with the gram-negative *Prevotella* genus in the gut, an observation that persisted three months after anthelminthic therapy [100]. Since *Prevotella* has been associated with mucosal inflammation along with Th17 polarisation in the gut and genital tract [175], this association could suggest a mechanism for enhanced HIV susceptibility at the mucosal interface. Interestingly, another study performed in India found a negative association between STH infections and intestinal *Lactobacillus* species [103]- also suggesting a shift toward a more proinflammatory microbial environment in the presence of STH, which could enhance HIV susceptibility.

## Lymphatic filariasis and HIV susceptibility

Lymphatic filariasis (LF), or elephantiasis, affects 37 African countries and is caused by the nematode *W. bancrofti* [176], a parasite that is transmitted by several different mosquito species. The *W. bancrofti* adult stage (a worm) resides in the lymphatic system of various organs, including the genital tract, while the larval stage (microfilariae) circulates in the blood [176]. Most LF-infected individuals remain asymptomatic, with a minority developing severe pathology, the hallmark of which is profound lymphoedema of affected organs [148, 176].

The first experimental evidence of LF-associated effects on HIV transmission was an *in vitro* study demonstrating that peripheral blood mononuclear cells from *W. bancrofti*-infected individuals sustained higher levels of HIV replication than cells from LF-free persons [177]. Subsequent cross-sectional studies in SSA reported conflicting associations between *W. bancrofti* and HIV prevalence [134, 178, 179]. However, a recent prospective study of >1000 initially HIV-uninfected Tanzanian adults of known LF infection status [180] demonstrated that LF-infected individuals were twice as likely to become HIV infected as their LF-uninfected peers after controlling for several sociobehavioural HIV risk factors [180]. Moreover, *W. bancrofti* infection has now been linked to elevated frequencies of circulating HLA-DR+ and HLA-DR+CD38+ as well as effector memory CD4 T cells, suggesting that the helminth-induced systemic CD4 T cell activation could be at least in part responsible for enhanced HIV susceptibility associated with this helminth [154].

## Schistosomiasis and HIV susceptibility

Schistosomiasis, also known as bilharzia or snail fever, is a neglected tropical disease transmitted via contact with contaminated freshwater and caused by flat worms of the genus *Schistosoma*. Schistosomiasis is highly prevalent across SSA [181] and in recent years schistosomiasis infection rates have increased due to dramatic environmental changes affecting water systems [182]. The mature schistosomes dwell in the blood vessels surrounding internal organs. The worms form couples consisting of a male and a female schistosome, in a permanent state of copulation and egg production [183]. Each egg secretes proteolytic enzymes that facilitate its migration into the internal organ lumen for subsequent excretion in either urine or feces (reviewed in [184, 185]).

A few weeks to months after primary infection with schistosomes, some individuals develop a systemic hypersensitivity reaction, known as Katayama fever, typically lasting 2-10 weeks [183]. The manifestations of this acute inflammatory response during the schistosomula migration include flu-like symptoms and hypereosinophilia, followed by abdominal symptoms caused by the settling of the mature worms [183]. Katayama fever is typically seen after primary infection in previously unexposed travelers to disease endemic regions, while in chronically exposed populations this acute response is not observed due to pre-existing tolerance induced during *in utero* exposure to helminth antigens [183]. After infection has been established, the tissue-trapped eggs are responsible for most of the damage to the host tissues. In particular, the eggs induce formation of pro-inflammatory granulomas, which become smaller as the infection enters a chronic phase [186]. These granulomas contain a variety of immune cells, including macrophages, dendritic cells, eosinophils, neutrophils, T and B cells, and their composition changes depending on disease severity [187].

### The two forms of schistosomiasis in SSA

The two main species responsible for schistosomiasis in SSA are: (i) *S. haematobium*, which is transmitted by *Bulinus* snails, and which resides within the perivesical veins where it causes genitourinary schistosomiasis; and (ii) *S. mansoni*, which is transmitted by *Biomphalaria* snails, and which dwells predominantly in the mesenteric veins where it causes intestinal and hepatic schistosomiasis [183] (Fig. 1). Fresh water bodies such as the River Nile and Lake Victoria in East Africa are typical sources of schistosomes, and schistosomiasis prevalence tends to be inversely proportional to the distance that a person lives from these water bodies [188]. The infection prevalence and intensity increase gradually with age, peaking around age 10-20 years and decreasing later in life, while high schistosomiasis burdens are typically seen only in a small proportion of infected individuals [183, 189]. The latter characteristics of schistosomiasis epidemiology appear to be guided by the exposed individuals’ water-contact patterns and anti-schistosomal immunity.

### *S. haematobium and* HIV susceptibility

*S. haematobium,* the cause of genitourinary schistosomiasis, is a WHO-recognized risk factor for HIV infection in women [190]. The prevalence of *S. haematobium* and HIV strongly correlate across SSA, after adjusting for multiple known HIV risk factors [191], while mathematical modelling indicates that exposure to the parasite elevates the odds of female HIV-positivity in Mozambique [192]. Notably, until recently *S. haematobium* was considered rare in South Africa, the country with the largest HIV epidemic in the world, but several recent surveys found substantial *S. haematobium* presence within multiple South African provinces, with an infection prevalence as high as 70% [193–196].

Cross-sectional epidemiological studies from Zimbabwe, Malawi and Tanzania reported that *S. haematobium*-infected women were are up to four-fold more likely to be HIV-infected compared to their peers without genitourinary schistosomiasis [189, 197, 198]. However, although a recent prospective study found *S. haematobium* infection to be associated with incident HIV transmission among Zambian couples [199], another recent study from East Africa showed no link between *S. haematobium* and HIV acquisition (unpublished data presented by Dr. Aaron Bochner and Dr. Ruanne Barnabas, University of Washington, [200]).

At the organ level, *S. haematobium* eggs can cause substantial damage to the pelvic tissues, involving the bladder, ureters, cervix and vagina. This leads to mucosal edema, open bleeding and an altered genital epithelium [201, 202], and directly increases HIV susceptibility through loss of epithelial integrity, increased vascularity and enhanced inflammation [189, 203]. Indeed, the host immune response to *S. haematobium* ova has been shown to recruit HIV target cells to the genital mucosa, including CD4+ T cells and macrophages [204].

Interestingly, recent reports describe distinct transcriptional profiles linked with *S. haematobium* infection in cross-sectional studies of whole blood- and cervical cytobrush-derived transcriptomes of individuals with and without schistosomiasis [205, 206]. While *S. haematobium*+ women also had reduced cervicovaginal IL-15 levels in these studies, the impact of this finding on HIV susceptibility is unclear, since IL-15 both stimulates anti-HIV immunity by enhancing the functionality of NK and CD8 T cells [207, 208] but also augments early HIV entry into CD4 T cells [209]. Nonetheless, these findings demonstrate that *S. haematobium* has substantial impact on both systemic and mucosal immunity.

Far less studied than in women, *S. haematobium* also causes male genital schistosomiasis (MGS), which may manifest as haematospermia and increased levels of seminal leukocytes in men [210]. However, it is thought that *S. haematobium* plays a lesser role in enhanced HIV susceptibility in men compared to women, because *S. haematobium*-affected male genital tissues are relatively proximal and so do not come into direct contact with HIV during condomless insertive penile sex [189]. Recent systematic reviews stress the urgent need for high quality clinical, randomized and epidemiological studies on MGS and HIV [189, 211].

### *S. mansoni* and HIV susceptibility

In most individuals *S. mansoni* infection is asymptomatic and does not lead to severe pathological sequelae. Like other schistosome species, *S. mansoni* are long-lived (up to 30 years) and are generally not cleared by the host immune system in the absence of anthelminthic therapy [212]. Post-mortem studies of *S. mansoni* infected individuals report the presence of extensive granulomatous inflammation, pseudopolyposis, ulcerations and bleeding in the colon and rectum [213, 214]. In some individuals the eggs trapped in the liver can cause hepatic schistosomiasis, the cause of abdominal organomegaly mainly in children and adolescents. Later in life, egg deposition in the periportal space can result in chronic hepatic schistosomiasis that causes portal hypertension and organomegaly [183].

#### The epidemiology of *S. mansoni*-HIV interaction

The overlap of *S. mansoni* infection with HIV prevalence in regions with very low *S. haematobium* infection has generated the hypothesis that *S. mansoni* might also increase HIV susceptibility. However, epidemiological studies performed so far have produced evidence both for and against this hypothesis. Studies performed in Tanzania reported that *S. mansoni*-infected women were six-fold more likely to be HIV-infected compared to their female peers without schistosomiasis [188], and subsequently a prospective study from the same group found that *S. mansoni*-infected women had a 2.8-fold increased risk of HIV acquisition [215]. Notably, these effects of *S. mansoni* on HIV acquisition in the Tanzanian studies were only seen in women, but not men [215, 216]. Furthermore, a study from Uganda reported that people with detectable antibodies against *S. mansoni* soluble egg antigens (SmSEA) were more likely to be HIV-positive compared to SmSEA-negative individuals [217], while a prospective study from Zambia found a trend to elevated HIV risk in women positive for *S.mansoni*-specific antibodies [199].

On the other hand, research in a separate cohort residing on the Ugandan shores of Lake Victoria did not find an association between prevalent or incident HIV and *S. mansoni* infection [218, 219]. However, this study recruited fewer females (88/200), and only 18 women (versus 84 men) were infected by *S. mansoni* [219]. Therefore, if *S. mansoni* only increases HIV susceptibility in women, the Ugandan studies may have been underpowered to detect an HIV association, and interestingly these studies did observe an association between *S. mansoni* treatment and decreased HIV prevalence [218, 219]. In addition, emerging data from a nested case-control study in Kenya and Uganda also show no association between *S. mansoni* infection and HIV risk (unpublished data presented by Dr. Aaron Bochner and Dr. Ruanne Barnabas, University of Washington [200]).

The reasons for these discrepant findings are not clear. In the earliest studies, region-specific HIV transmission dynamics could have played a role in the different outcomes seen by Tanzanian and Ugandan researchers, since the HIV prevalence in Uganda (17.3%) was about 3-fold higher than Tanzania (5.6-6.1%). Furthermore, neither the early Uganda or Tanzania-based studies assessed injectable contraceptive use, which varies substantially across East African countries [220, 221], is linked with both altered genital immunology [222] and HIV acquisition [3], and which was inversely correlated with *S. mansoni* infection in Ugandan women [223]. However, more recent studies that do control for these parameters still generate conflicting results, making it important to consider biological mechanisms by which *S. mansoni* infection might enhance HIV susceptibility in the FGT.

#### The biology of *S. mansoni*-HIV interaction

It is not well understood how *S. mansoni* infection could increase HIV susceptibility, and why this effect appears only in women. Acute *S. mansoni* infection in rhesus macaques increases their susceptibility to a rectal SHIV challenge, with mucosal infection requiring 17-fold less virus compared to schistosoma-free animals, but vaginal challenge was not performed in these studies [224, 225]. Furthermore, individuals with intestinal schistosomiasis exhibited elevated levels of TLR2 and 4 expressing B cells [155] and high levels of blood LPS [155, 157], indicating helminth-induced bacterial translocation due to decreased integrity of the gut mucosal barrier.

While it is logical that intestinal schistosomiasis would have a direct effect on HIV transmission after sexual exposure in the rectal mucosa, helminth-induced gut mucosal inflammation could theoretically involve other mucosal sites (such as the female lower genital tract) through activation of the common mucosal immune system [79]. In keeping with this, *S. mansoni*-infected women with a higher parasite burden demonstrated elevated expression of the mucosal homing integrin α4β7 on blood CD4+ T cells [226], which would be expected to home these CD4 cells to the gut and cervical mucosa. However, this integrin does not appear to home T cells to the foreskin, the predominant site of HIV acquisition in heterosexual men from SSA, since the predominant integrin expressed on T cells in foreskin tissues is cutaneous lymphocyte antigen (CLA) [48]. The latter could at least partly explain the differential impact of *S. mansoni* infection on HIV susceptibility in women versus men.

Curiously, a recent study found cervical gene expression to be unaltered by *S. mansoni* infection [206], however transcriptional analysis in this study was done using unfractionated cervical samples, which would detect changes in the most common mucosal cells (especially epithelial cells) but might not detect changes occurring at the level of less frequent mucosal cell subsets, such as CD4+ T cells.

#### HIV target cells in *S. mansoni* infection

At the cellular level, schistosomiasis has been associated with increased expression of CD4+ T cell parameters that enhance HIV susceptibility, including increased CCR5+ expression and a Th17 phenotype. Secor and colleagues reported elevated expression of CCR5 and CXCR4 on circulating CD4 T cells of *S. mansoni*-infected Kenyan men, and their expression dropped after schistosomiasis treatment [227]. Furthermore, studies in murine models indicate that the parasite-driven granuloma formation is mediated by Th17 cells. Specifically, schistosomiasis immunopathology in internal organs is strongly associated with Th17-inducing cytokines such as IL-23, and Th17-produced cytokines such as IL-17 and IL-22 [167]. Th17 cells appear to control granulomatous inflammation by regulating neutrophil infiltration [167]. Interestingly, the profiles of circulating Th17 cells have been shown to correlate well with those seen in tissues of *S. mansoni*-infected mice [168] and Th17 cells were present at higher frequencies in the blood of *S. mansoni*-infected Ugandans [228]. Given that Th17 cells are a primary target of HIV [71], the elevated levels of these cells may be important contributors to enhanced HIV acquisition in *S. mansoni*-infected individuals.

#### Evidence for direct urogenital effects of “intestinal” *S. mansoni* infection

The recent findings of elevated HIV acquisition in women with *S. mansoni* infection in some studies raise the possibility that this helminth infection may have direct urogenital effects [215]. Although classically considered a mesenteric infection, early autopsy studies in *S. mansoni*-infected individuals found that 24% of all eggs were lodged in the urogenital tract [213]. Further, studies in Tanzanian women found *S. mansoni* eggs in cervical biopsies to be associated with cervical lesions [201]. Based on several other reports [229, 230], Feldmeier and colleagues postulated that due to both host and parasite-dependent factors, up to 27% of women with intestinal schistosomiasis show pathological signs due to *S. mansoni* eggs trapped in their urogenital tract [231]. Therefore, Downs and colleagues proposed that the effects of *S. mansoni* on HIV susceptibility could be attributed to the direct effects of helminth eggs on the urogenital mucosa [215].

This mechanism could thus explain the sex-biased effects of *S. mansoni*, due to the differences in the anatomical structure of the genital tract in men versus women. Specifically, the genitourinary organs most affected by the eggs of *S. haematobium* and *S. mansoni* in men are the prostate and seminal vesicles [213, 232, 233], but not the penis, the primary site of HIV acquisition in heterosexual men [53]. In keeping with this, schistosome-infected women also shed fewer parasite eggs than schistosome+ men for a given worm burden [234], suggesting that schistosome eggs in women are more frequently trapped inside the body compared to men. This finding also has implications for the overall levels of inflammation and HIV susceptibility: at a similar worm burden, more trapped eggs would translate into elevated mucosal inflammation and HIV susceptibility in schistosome+ women compared to men.

## Could the treatment of endemic infections reduce HIV susceptibility?

If endemic infections do elevate HIV susceptibility, then their treatment and/or prophylaxis could be an effective addition to the HIV prevention toolbox. While a meta-analysis of studies in HIV-infected individuals indicated substantial changes in HIV blood viral load after the treatment of co-infections [8]. However, data about any effects of endemic infection treatment on HIV susceptibility are lacking, due to the paucity of prospective studies [189].

Deworming could theoretically reduce HIV susceptibility by lowering helminth-induced inflammation in tissues, lifting systemic immune suppression and down-regulating HIV co-receptor expression. For instance, schistosomiasis therapy reduces circulating Tregs and innate immune cells involved in granulomatous inflammation [235, 236], thus lifting suppression of antiviral immunity and reducing HIV infection-enhancing inflammation (although removal of Tregs might also favour HIV susceptibility by increasing the number of activated cells [118, 237]. Furthermore, *S. mansoni* treatment in Kenyan men decreased HIV co-receptor CCR5/CXCR4 density on circulating CD4 T cells [227] and a reduction of CCR5 expression was reported after treatment of *Trichuris* in Tanzania [153]. Similar observations were made in South African women treated for *S. haematobium*, whereby CCR5 expression by CD4+ T cells decreased significantly in blood and reductions were seen for monocyte CCR5 expression in both blood and the cervix 7-8 months after treatment [203].

Based on the epidemiological evidence of *S. haematobium*-amplified HIV transmission, mathematical modeling forecasts that treatment of genitourinary *S. haematobium* infections in school-age children could be a highly cost-effective intervention for preventing HIV infection in schistosome-endemic areas [238, 239]. According to these models, over a decade of annual praziquantel administration, an amount of $52-260 would be spent per every HIV case averted- a more cost-effective HIV prevention strategy compared to STI treatment or male circumcision. Given that *S. mansoni* infection has been associated with an HIV risk similar to that seen for *S. haematobium* [189] and that in *S. mansoni*-endemic Uganda a history of schistosomiasis treatment was linked to lower HIV risk [218, 219], it is plausible that “intestinal” *S. mansoni* infection treatment would also be a cost-effective strategy for HIV prevention.

In keeping with the earlier studies, our recent work [226] provides support for future clinical studies of *S. mansoni* treatment as an HIV prevention strategy. Specifically, we found that *S. mansoni* treatment resulted in an over two- fold reduction of *ex vivo* HIV entry into genital and blood CD4 T cells, but surprisingly this reduced virus entry after praziquantel therapy was accompanied by transient immune activation in the cervix and blood. Traditionally, immune activation is thought to elevate HIV susceptibility [59], and to increase HIV entry into CD4 T cells [240]. However, in some contexts immune activation can accompany a strong antiviral immune response incapacitating multiple HIV infection stages, from cellular entry to production of virus progeny [241–243]. Based on these studies, we hypothesized that *S. mansoni* treatment resulted in the induction of antiviral signaling. Subsequent experiments provided evidence of elevated mucosal IFN-α2a and a systemic transcriptomic signature of interferon signaling induction after *S. mansoni* treatment. Remarkably, untreated *S. mansoni* infection was associated with antiviral gene down-regulation and praziquantel therapy partially reversed this helminth-induced immune suppression [226].

Lastly, the effects of chronic infections can be long-lasting even after successful clearance of parasites, as observed, for example, after *S. haematobium* treatment, whereby parasite DNA was still detectable in the genital tract along with anatomical abnormalities six months post-deworming [244]. This means that it will be important to choose an appropriate time-frame for future studies that aim to investigate the effects of deworming on HIV susceptibility.

## Conclusion

HIV continues to exert a substantial toll on the lives of people in SSA, and recent evidence suggests that there is considerable interaction between parasitic infections and HIV transmission in this region. Previously, the impact of endemic infections on HIV transmission had been explored mainly in the context of co-infection in HIV+ individuals. In this review we summarized the evidence for and against the effects of parasitic infections on HIV susceptibility in HIV-uninfected individuals. The paucity of data in this field, and the contradictory nature of the results from the few studies that have been performed, emphasizes the need for well-designed clinical trials to investigate the effects of parasitic infections and their treatment on HIV incidence in endemic communities. Ultimately, effective control of parasitic infections might not only reduce widespread morbidity directly caused by these infections, but might also reduce HIV transmission among the millions of at-risk individuals exposed to the endemic infections in SSA.

## Data Availability

Not applicable

## References

[1] UNAIDS. UNAIDS Global HIV and AIDS statistics [Internet]. 2018 [Accessed 10 May 2019]. Available from: http://www.unaids.org/en/resources/fact-sheet.

[2] Boily MC, Baggaley RF, Wang L, Masse B, White RG, Hayes RJ (2009). Heterosexual risk of HIV-1 infection per sexual act: systematic review and meta-analysis of observational studies. Lancet Infect Dis.

[3] Ralph LJ, McCoy SI, Shiu K, Padian NS (2015). Hormonal contraceptive use and women's risk of HIV acquisition: a meta-analysis of observational studies. Lancet Infect Dis.

[4] Low N, Chersich MF, Schmidlin K, Egger M, Francis SC, van de Wijgert JH (2011). Intravaginal practices, bacterial vaginosis, and HIV infection in women: individual participant data meta-analysis. PLoS Med.

[5] Rottingen JA, Cameron DW, Garnett GP (2001). A systematic review of the epidemiologic interactions between classic sexually transmitted diseases and HIV: how much really is known?. Sex Transm Dis.

[6] Looker KJ, Elmes JAR, Gottlieb SL, Schiffer JT, Vickerman P, Turner KME (2017). Effect of HSV-2 infection on subsequent HIV acquisition: an updated systematic review and meta-analysis. Lancet Infect Dis.

[7] Brown M, Mawa PA, Kaleebu P, Elliott AM (2006). Helminths and HIV infection: epidemiological observations on immunological hypotheses. Parasite Immunol.

[8] Modjarrad K, Vermund SH (2010). Effect of treating co-infections on HIV-1 viral load: a systematic review. Lancet Infect Dis.

[9] Harms G, Feldmeier H (2002). HIV infection and tropical parasitic diseases - deleterious interactions in both directions?. Tropical Med Int Health.

[10] Quinn TC, Wawer MJ, Sewankambo N, Serwadda D, Li C, Wabwire-Mangen F (2000). Viral load and heterosexual transmission of human immunodeficiency virus type 1. Rakai Project Study Group. N Engl J Med.

[11] Molyneux DH, Hotez PJ, Fenwick A (2005). “Rapid-impact interventions”: how a policy of integrated control for Africa’s neglected tropical diseases could benefit the poor. PLoS Med.

[12] Hotez PJ, Molyneux DH, Fenwick A, Ottesen E, Ehrlich Sachs S, Sachs JD (2006). Incorporating a rapid-impact package for neglected tropical diseases with programs for HIV/AIDS, tuberculosis, and malaria. PLoS Med.

[13] Hotez PJ, Fenwick A, Ray SE, Hay SI, Molyneux DH (2018). “Rapid impact” 10 years after: The first “decade” (2006-2016) of integrated neglected tropical disease control. PLoS Negl Trop Dis.

[14] Hotez PJ, Harrison W, Fenwick A, Bustinduy AL, Ducker C, Sabina Mbabazi P (2019). Female genital schistosomiasis and HIV/AIDS: Reversing the neglect of girls and women. PLoS Negl Trop Dis.

[15] Hladik F, McElrath MJ (2008). Setting the stage: host invasion by HIV. Nat Rev Immunol.

[16] Powers KA, Poole C, Pettifor AE, Cohen MS (2008). Rethinking the heterosexual infectivity of HIV-1: a systematic review and meta-analysis. Lancet Infect Dis.

[17] Hughes JP, Baeten JM, Lingappa JR, Magaret AS, Wald A, de Bruyn G (2012). Determinants of per-coital-act HIV-1 infectivity among African HIV-1-serodiscordant couples. J Infect Dis.

[18] Gray RH, Wawer MJ, Brookmeyer R, Sewankambo NK, Serwadda D, Wabwire-Mangen F (2001). Probability of HIV-1 transmission per coital act in monogamous, heterosexual, HIV-1-discordant couples in Rakai, Uganda. Lancet.

[19] Fideli US, Allen SA, Musonda R, Trask S, Hahn BH, Weiss H (2001). Virologic and immunologic determinants of heterosexual transmission of human immunodeficiency virus type 1 in Africa. AIDS Res Hum Retrovir.

[20] Allen S, Serufilira A, Bogaerts J, Van de Perre P, Nsengumuremyi F, Lindan C (1992). Confidential HIV testing and condom promotion in Africa. Impact on HIV and gonorrhea rates. JAMA..

[21] Gouws Eleanor, Cuchi Paloma (2012). Focusing the HIV response through estimating the major modes of HIV transmission: a multi-country analysis. Sexually Transmitted Infections.

[22] Ramjee G, Daniels B (2013). Women and HIV in Sub-Saharan Africa. AIDS Res Ther.

[23] Glynn JR, Carael M, Auvert B, Kahindo M, Chege J, Musonda R (2001). Why do young women have a much higher prevalence of HIV than young men? A study in Kisumu, Kenya and Ndola, Zambia. AIDS..

[24] Magadi MA (2011). Understanding the gender disparity in HIV infection across countries in sub-Saharan Africa: evidence from the Demographic and Health Surveys. Sociol Health Illn.

[25] Kumamoto Y, Iwasaki A (2012). Unique features of antiviral immune system of the vaginal mucosa. Curr Opin Immunol.

[26] Zhang X, Mozeleski B, Lemoine S, Deriaud E, Lim A, Zhivaki D (2014). CD4 T cells with effector memory phenotype and function develop in the sterile environment of the fetus. Sci Transl Med.

[27] Stieh DJ, Maric D, Kelley ZL, Anderson MR, Hattaway HZ, Beilfuss BA (2014). Vaginal challenge with an SIV-based dual reporter system reveals that infection can occur throughout the upper and lower female reproductive tract. PLoS Pathog.

[28] Deleage C, Immonen TT, Fennessey CM, Reynaldi A, Reid C, Newman L (2019). Defining early SIV replication and dissemination dynamics following vaginal transmission. Sci Adv.

[29] Haase AT (2010). Targeting early infection to prevent HIV-1 mucosal transmission. Nature..

[30] Haase AT (2005). Perils at mucosal front lines for HIV and SIV and their hosts. Nat Rev Immunol.

[31] Carias AM, McCoombe S, McRaven M, Anderson M, Galloway N, Vandergrift N (2013). Defining the interaction of HIV-1 with the mucosal barriers of the female reproductive tract. J Virol.

[32] Miller CJ, Li Q, Abel K, Kim EY, Ma ZM, Wietgrefe S (2005). Propagation and dissemination of infection after vaginal transmission of simian immunodeficiency virus. J Virol.

[33] Hu J, Gardner MB, Miller CJ (2000). Simian immunodeficiency virus rapidly penetrates the cervicovaginal mucosa after intravaginal inoculation and infects intraepithelial dendritic cells. J Virol.

[34] Zhang Z, Schuler T, Zupancic M, Wietgrefe S, Staskus KA, Reimann KA (1999). Sexual transmission and propagation of SIV and HIV in resting and activated CD4+ T cells. Science..

[35] Barouch DH, Ghneim K, Bosche WJ, Li Y, Berkemeier B, Hull M (2016). Rapid Inflammasome Activation following Mucosal SIV Infection of Rhesus Monkeys. Cell..

[36] Haase AT (2011). Early events in sexual transmission of HIV and SIV and opportunities for interventions. Annu Rev Med.

[37] Thigpen MC, Kebaabetswe PM, Paxton LA, Smith DK, Rose CE, Segolodi TM (2012). Antiretroviral preexposure prophylaxis for heterosexual HIV transmission in Botswana. N Engl J Med.

[38] Baeten JM, Donnell D, Ndase P, Mugo NR, Campbell JD, Wangisi J (2012). Antiretroviral prophylaxis for HIV prevention in heterosexual men and women. N Engl J Med.

[39] Veazey RS, Pilch-Cooper HA, Hope TJ, Alter G, Carias AM, Sips M (2016). Prevention of SHIV transmission by topical IFN-beta treatment. Mucosal Immunol.

[40] Deruaz M, Murooka TT, Ji S, Gavin MA, Vrbanac VD, Lieberman J (2017). Chemoattractant-mediated leukocyte trafficking enables HIV dissemination from the genital mucosa. JCI Insight.

[41] Li Q, Estes JD, Schlievert PM, Duan L, Brosnahan AJ, Southern PJ (2009). Glycerol monolaurate prevents mucosal SIV transmission. Nature..

[42] Lajoie J, Mwangi L, Fowke KR (2017). Preventing HIV infection without targeting the virus: how reducing HIV target cells at the genital tract is a new approach to HIV prevention. AIDS Res Ther.

[43] Cohen YZ, Caskey M (2018). Broadly neutralizing antibodies for treatment and prevention of HIV-1 infection. Curr Opin HIV AIDS.

[44] McMichael AJ, Koff WC (2014). Vaccines that stimulate T cell immunity to HIV-1: the next step. Nat Immunol.

[45] Sennepin A, Real F, Duvivier M, Ganor Y, Henry S, Damotte D (2017). The Human Penis Is a Genuine Immunological Effector Site. Front Immunol.

[46] McCoombe SG, Short RV (2006). Potential HIV-1 target cells in the human penis. AIDS..

[47] Fischetti L, Barry SM, Hope TJ, Shattock RJ (2009). HIV-1 infection of human penile explant tissue and protection by candidate microbicides. AIDS..

[48] Galiwango RM, Yegorov S, Joag V, Prodger J, Shahabi K, Huibner S (2019). Characterization of CD4(+) T cell subsets and HIV susceptibility in the inner and outer foreskin of Ugandan men. Am J Reprod Immunol.

[49] Prodger JL, Hirbod T, Gray R, Kigozi G, Nalugoda F, Galiwango R (2014). HIV Infection in Uncircumcised Men Is Associated With Altered CD8 T-cell Function But Normal CD4 T-cell Numbers in the Foreskin. J Infect Dis.

[50] Prodger JL, Gray R, Kigozi G, Nalugoda F, Galiwango R, Nehemiah K (2012). Impact of asymptomatic Herpes simplex virus-2 infection on T cell phenotype and function in the foreskin. AIDS..

[51] Prodger JL, Gray R, Kigozi G, Nalugoda F, Galiwango R, Hirbod T (2012). Foreskin T-cell subsets differ substantially from blood with respect to HIV co-receptor expression, inflammatory profile, and memory status. Mucosal Immunol.

[52] Prodger JL, Hirbod T, Kigozi G, Nalugoda F, Reynolds SJ, Galiwango R (2014). Immune correlates of HIV exposure without infection in foreskins of men from Rakai, Uganda. Mucosal Immunol.

[53] Anderson D, Politch JA, Pudney J (2011). HIV infection and immune defense of the penis. Am J Reprod Immunol.

[54] Kigozi G, Wawer M, Ssettuba A, Kagaayi J, Nalugoda F, Watya S (2009). Foreskin surface area and HIV acquisition in Rakai, Uganda (size matters). AIDS..

[55] Prodger JL, Kaul R (2017). The biology of how circumcision reduces HIV susceptibility: broader implications for the prevention field. AIDS Res Ther.

[56] Morhason-Bello IO, Kabakama S, Baisley K, Francis SC, Watson-Jones D (2019). Reported oral and anal sex among adolescents and adults reporting heterosexual sex in sub-Saharan Africa: a systematic review. Reprod Health.

[57] Baggaley RF, White RG, Boily MC (2010). HIV transmission risk through anal intercourse: systematic review, meta-analysis and implications for HIV prevention. Int J Epidemiol.

[58] Kelley CF, Kraft CS, de Man TJ, Duphare C, Lee HW, Yang J (2017). The rectal mucosa and condomless receptive anal intercourse in HIV-negative MSM: implications for HIV transmission and prevention. Mucosal Immunol.

[59] McKinnon LR, Kaul R (2012). Quality and quantity: mucosal CD4+ T cells and HIV susceptibility. Curr Opin HIV AIDS.

[60] Kariuki SM, Selhorst P, Arien KK, Dorfman JR (2017). The HIV-1 transmission bottleneck. Retrovirology..

[61] Grivel Jean-Charles, Shattock Robin J, Margolis Leonid B (2010). Selective transmission of R5 HIV-1 variants: where is the gatekeeper?. Journal of Translational Medicine.

[62] Saba E, Grivel JC, Vanpouille C, Brichacek B, Fitzgerald W, Margolis L (2010). HIV-1 sexual transmission: early events of HIV-1 infection of human cervico-vaginal tissue in an optimized ex vivo model. Mucosal Immunol.

[63] Carnathan DG, Wetzel KS, Yu J, Lee ST, Johnson BA, Paiardini M (2015). Activated CD4+CCR5+ T cells in the rectum predict increased SIV acquisition in SIVGag/Tat-vaccinated rhesus macaques. Proc Natl Acad Sci U S A.

[64] Koning FA, Otto SA, Hazenberg MD, Dekker L, Prins M, Miedema F (2005). Low-level CD4+ T cell activation is associated with low susceptibility to HIV-1 infection. J Immunol.

[65] Chattopadhyay PK, Roederer M (2010). Good cell, bad cell: flow cytometry reveals T-cell subsets important in HIV disease. Cytometry A.

[66] Cibrian D, Sanchez-Madrid F (2017). CD69: from activation marker to metabolic gatekeeper. Eur J Immunol.

[67] Gosselin A, Monteiro P, Chomont N, Diaz-Griffero F, Said EA, Fonseca S (2010). Peripheral blood CCR4+CCR6+ and CXCR3+CCR6+CD4+ T cells are highly permissive to HIV-1 infection. J Immunol.

[68] Alvarez Y, Tuen M, Shen G, Nawaz F, Arthos J, Wolff MJ (2013). Preferential HIV infection of CCR6+ Th17 cells is associated with higher levels of virus receptor expression and lack of CCR5 ligands. J Virol.

[69] Sallusto F, Zielinski CE, Lanzavecchia A (2012). Human Th17 subsets. Eur J Immunol.

[70] Annunziato F, Cosmi L, Liotta F, Maggi E, Romagnani S (2012). Defining the human T helper 17 cell phenotype. Trends Immunol.

[71] Stieh DJ, Matias E, Xu H, Fought AJ, Blanchard JL, Marx PA (2016). Th17 Cells Are Preferentially Infected Very Early after Vaginal Transmission of SIV in Macaques. Cell Host Microbe.

[72] McKinnon LR, Nyanga B, Chege D, Izulla P, Kimani M, Huibner S (2011). Characterization of a human cervical CD4+ T cell subset coexpressing multiple markers of HIV susceptibility. J Immunol.

[73] Rodriguez-Garcia M, Barr FD, Crist SG, Fahey JV, Wira CR (2014). Phenotype and susceptibility to HIV infection of CD4+ Th17 cells in the human female reproductive tract. Mucosal Immunol.

[74] Berlin C, Berg EL, Briskin MJ, Andrew DP, Kilshaw PJ, Holzmann B (1993). Alpha 4 beta 7 integrin mediates lymphocyte binding to the mucosal vascular addressin MAdCAM-1. Cell..

[75] Arthos J, Cicala C, Nawaz F, Byrareddy SN, Villinger F, Santangelo PJ (2018). The Role of Integrin alpha4beta7 in HIV Pathogenesis and Treatment. Curr HIV/AIDS Rep.

[76] Humphries JD, Byron A, Humphries MJ (2006). Integrin ligands at a glance. J Cell Sci.

[77] Holmgren J, Czerkinsky C (2005). Mucosal immunity and vaccines. Nat Med.

[78] Bienenstock J, McDermott M, Befus D, O'Neill M (1978). A common mucosal immunologic system involving the bronchus, breast and bowel. Adv Exp Med Biol.

[79] Gill N, Wlodarska M, Finlay BB (2010). The future of mucosal immunology: studying an integrated system-wide organ. Nat Immunol.

[80] Sato A, Suwanto A, Okabe M, Sato S, Nochi T, Imai T (2014). Vaginal memory T cells induced by intranasal vaccination are critical for protective T cell recruitment and prevention of genital HSV-2 disease. J Virol.

[81] Gallichan WS, Woolstencroft RN, Guarasci T, McCluskie MJ, Davis HL, Rosenthal KL (2001). Intranasal immunization with CpG oligodeoxynucleotides as an adjuvant dramatically increases IgA and protection against herpes simplex virus-2 in the genital tract. J Immunol.

[82] Gillgrass AE, Tang VA, Towarnicki KM, Rosenthal KL, Kaushic C (2005). Protection against genital herpes infection in mice immunized under different hormonal conditions correlates with induction of vagina-associated lymphoid tissue. J Virol.

[83] Tan X, Sande JL, Pufnock JS, Blattman JN, Greenberg PD (2011). Retinoic acid as a vaccine adjuvant enhances CD8+ T cell response and mucosal protection from viral challenge. J Virol.

[84] Perciani CT, Jaoko W, Farah B, Ostrowski MA, Anzala O, MacDonald KS (2018). alphaEbeta7, alpha4beta7 and alpha4beta1 integrin contributions to T cell distribution in blood, cervix and rectal tissues: Potential implications for HIV transmission. PLoS One.

[85] Jacobs W, Van Marck E (1998). Adhesion and co-stimulatory molecules in the pathogenesis of hepatic and intestinal schistosomiasis mansoni. Mem Inst Oswaldo Cruz.

[86] Chege D, Higgins SJ, McDonald CR, Shahabi K, Huibner S, Kain T, et al. Murine Plasmodium chabaudi malaria increases mucosal immune activation and the expression of putative HIV susceptibility markers in the gut and genital mucosa. J Acquir Immune Defic Syndr. 2013. 10.1097/QAI.0000000000000056 24256632

[87] Burgener A, McGowan I, Klatt NR (2015). HIV and mucosal barrier interactions: consequences for transmission and pathogenesis. Curr Opin Immunol.

[88] Eastment MC, McClelland RS (2018). Vaginal microbiota and susceptibility to HIV. AIDS..

[89] Cherpes TL, Meyn LA, Krohn MA, Lurie JG, Hillier SL (2003). Association between acquisition of herpes simplex virus type 2 in women and bacterial vaginosis. Clin Infect Dis.

[90] McClelland RS, Lingappa JR, Srinivasan S, Kinuthia J, John-Stewart GC, Jaoko W (2018). Evaluation of the association between the concentrations of key vaginal bacteria and the increased risk of HIV acquisition in African women from five cohorts: a nested case-control study. Lancet Infect Dis.

[91] Gosmann C, Anahtar MN, Handley SA, Farcasanu M, Abu-Ali G, Bowman BA (2017). Lactobacillus-Deficient Cervicovaginal Bacterial Communities Are Associated with Increased HIV Acquisition in Young South African Women. Immunity..

[92] Martin HL, Richardson BA, Nyange PM, Lavreys L, Hillier SL, Chohan B (1999). Vaginal lactobacilli, microbial flora, and risk of human immunodeficiency virus type 1 and sexually transmitted disease acquisition. J Infect Dis.

[93] Klatt NR, Cheu R, Birse K, Zevin AS, Perner M, Noel-Romas L (2017). Vaginal bacteria modify HIV tenofovir microbicide efficacy in African women. Science..

[94] Ivashkiv LB, Donlin LT (2014). Regulation of type I interferon responses. Nat Rev Immunol.

[95] Doyle T, Goujon C, Malim MH (2015). HIV-1 and interferons: who's interfering with whom?. Nat Rev Microbiol.

[96] Iyer SS, Bibollet-Ruche F, Sherrill-Mix S, Learn GH, Plenderleith L, Smith AG (2017). Resistance to type 1 interferons is a major determinant of HIV-1 transmission fitness. Proc Natl Acad Sci U S A.

[97] Obajemu AA, Rao N, Dilley KA, Vargas JM, Sheikh F, Donnelly RP (2017). IFN-lambda4 Attenuates Antiviral Responses by Enhancing Negative Regulation of IFN Signaling. J Immunol.

[98] Kotenko SV, Durbin JE (2017). Contribution of type III interferons to antiviral immunity: location, location, location. J Biol Chem.

[99] Rihn SJ, Foster TL, Busnadiego I, Aziz MA, Hughes J, Neil SJ (2017). The Envelope Gene of Transmitted HIV-1 Resists a Late Interferon Gamma-Induced Block. J Virol.

[100] Kay GL, Millard A, Sergeant MJ, Midzi N, Gwisai R, Mduluza T (2015). Differences in the Faecal Microbiome in Schistosoma haematobium Infected Children vs. Uninfected Children. PLoS Negl Trop Dis.

[101] Zhao Y, Yang S, Li B, Li W, Wang J, Chen Z (2019). Alterations of the Mice Gut Microbiome via Schistosoma japonicum Ova-Induced Granuloma. Front Microbiol.

[102] Martin I, Djuardi Y, Sartono E, Rosa BA, Supali T, Mitreva M (2018). Dynamic changes in human-gut microbiome in relation to a placebo-controlled anthelminthic trial in Indonesia. PLoS Negl Trop Dis.

[103] Huwe T, Prusty BK, Ray A, Lee S, Ravindran B, Michael E (2019). Interactions between Parasitic Infections and the Human Gut Microbiome in Odisha, India. Am J Trop Med Hyg.

[104] Easton AV, Quinones M, Vujkovic-Cvijin I, Oliveira RG, Kepha S, Odiere MR (2019). The Impact of Anthelmintic Treatment on Human Gut Microbiota Based on Cross-Sectional and Pre- and Postdeworming Comparisons in Western Kenya. MBio.

[105] Ajibola O, Rowan AD, Ogedengbe CO, Mshelia MB, Cabral DJ, Eze AA (2019). Urogenital schistosomiasis is associated with signatures of microbiome dysbiosis in Nigerian adolescents. Sci Rep.

[106] Webb LM, Lundie RJ, Borger JG, Brown SL, Connor LM, Cartwright AN (2017). Type I interferon is required for T helper (Th) 2 induction by dendritic cells. EMBO J.

[107] Aksoy E, Zouain CS, Vanhoutte F, Fontaine J, Pavelka N, Thieblemont N (2005). Double-stranded RNAs from the helminth parasite Schistosoma activate TLR3 in dendritic cells. J Biol Chem.

[108] Trottein F, Pavelka N, Vizzardelli C, Angeli V, Zouain CS, Pelizzola M (2004). A type I IFN-dependent pathway induced by Schistosoma mansoni eggs in mouse myeloid dendritic cells generates an inflammatory signature. J Immunol.

[109] Nazli A, Dizzell S, Zahoor MA, Ferreira VH, Kafka J, Woods MW, et al. Interferon-beta induced in female genital epithelium by HIV-1 glycoprotein 120 via Toll-like-receptor 2 pathway acts to protect the mucosal barrier. Cell Mol Immunol. 2018. 10.1038/cmi.2017.168 PMC6355787 29553138

[110] Arnold KB, Burgener A, Birse K, Romas L, Dunphy LJ, Shahabi K (2016). Increased levels of inflammatory cytokines in the female reproductive tract are associated with altered expression of proteases, mucosal barrier proteins, and an influx of HIV-susceptible target cells. Mucosal Immunol.

[111] Levinson P, Kaul R, Kimani J, Ngugi E, Moses S, MacDonald KS (2009). Levels of innate immune factors in genital fluids: association of alpha defensins and LL-37 with genital infections and increased HIV acquisition. AIDS..

[112] Masson L, Passmore JA, Liebenberg LJ, Werner L, Baxter C, Arnold KB (2015). Genital inflammation and the risk of HIV acquisition in women. Clin Infect Dis.

[113] Naranbhai V, Abdool Karim SS, Altfeld M, Samsunder N, Durgiah R, Sibeko S (2012). Innate immune activation enhances hiv acquisition in women, diminishing the effectiveness of tenofovir microbicide gel. J Infect Dis.

[114] Kahle EM, Bolton M, Hughes JP, Donnell D, Celum C, Lingappa JR (2015). Plasma cytokine levels and risk of HIV type 1 (HIV-1) transmission and acquisition: a nested case-control study among HIV-1-serodiscordant couples. J Infect Dis.

[115] Lajoie J, Juno J, Burgener A, Rahman S, Mogk K, Wachihi C (2012). A distinct cytokine and chemokine profile at the genital mucosa is associated with HIV-1 protection among HIV-exposed seronegative commercial sex workers. Mucosal Immunol.

[116] Chege D, Chai Y, Huibner S, Kain T, Wachihi C, Kimani M (2012). Blunted IL17/IL22 and pro-inflammatory cytokine responses in the genital tract and blood of HIV-exposed, seronegative female sex workers in Kenya. PLoS One.

[117] Card CM, Rutherford WJ, Ramdahin S, Yao X, Kimani M, Wachihi C (2012). Reduced cellular susceptibility to in vitro HIV infection is associated with CD4+ T cell quiescence. PLoS One.

[118] Card CM, McLaren PJ, Wachihi C, Kimani J, Plummer FA, Fowke KR (2009). Decreased immune activation in resistance to HIV-1 infection is associated with an elevated frequency of CD4(+)CD25(+)FOXP3(+) regulatory T cells. J Infect Dis.

[119] Suy A, Castro P, Nomdedeu M, Garcia F, Lopez A, Fumero E (2007). Immunological profile of heterosexual highly HIV-exposed uninfected individuals: predominant role of CD4 and CD8 T-cell activation. J Infect Dis.

[120] Biasin M, Caputo SL, Speciale L, Colombo F, Racioppi L, Zagliani A (2000). Mucosal and systemic immune activation is present in human immunodeficiency virus-exposed seronegative women. J Infect Dis.

[121] Kyongo JK, Crucitti T, Menten J, Hardy L, Cools P, Michiels J (2015). Cross-Sectional Analysis of Selected Genital Tract Immunological Markers and Molecular Vaginal Microbiota in Sub-Saharan African Women, with Relevance to HIV Risk and Prevention. Clin Vaccine Immunol.

[122] Francis SC, Hou Y, Baisley K, van de Wijgert J, Watson-Jones D, Ao TT (2016). Immune Activation in the Female Genital Tract: Expression Profiles of Soluble Proteins in Women at High Risk for HIV Infection. PLoS One.

[123] Masson L, Mlisana K, Little F, Werner L, Mkhize NN, Ronacher K (2014). Defining genital tract cytokine signatures of sexually transmitted infections and bacterial vaginosis in women at high risk of HIV infection: a cross-sectional study. Sex Transm Infect.

[124] Joag V, Sivro A, Yende-Zuma N, Imam H, Samsunder N, Abdool Karim Q (2018). Ex vivo HIV entry into blood CD4+ T cells does not predict heterosexual HIV acquisition in women. PLoS One.

[125] Liebenberg LJ, Masson L, Arnold KB, McKinnon LR, Werner L, Proctor E (2017). Genital-Systemic Chemokine Gradients and the Risk of HIV Acquisition in Women. J Acquir Immune Defic Syndr.

[126] Promadej-Lanier N, Hanson DL, Srinivasan P, Luo W, Adams DR, Guenthner PC (2010). Resistance to Simian HIV infection is associated with high plasma interleukin-8, RANTES and Eotaxin in a macaque model of repeated virus challenges. J Acquir Immune Defic Syndr.

[127] Bhatt S, Weiss DJ, Cameron E, Bisanzio D, Mappin B, Dalrymple U (2015). The effect of malaria control on Plasmodium falciparum in Africa between 2000 and 2015. Nature..

[128] Miller LH, Ackerman HC, Su XZ, Wellems TE (2013). Malaria biology and disease pathogenesis: insights for new treatments. Nat Med.

[129] WHO (2015). World Malaria Report 2015.

[130] Bell D, Wongsrichanalai C, Barnwell JW (2006). Ensuring quality and access for malaria diagnosis: how can it be achieved?. Nat Rev Microbiol.

[131] Ohrt C, Purnomo, Sutamihardja MA, Tang D, Kain KC (2002). Impact of microscopy error on estimates of protective efficacy in malaria-prevention trials. J Infect Dis.

[132] Yegorov S, Galiwango RM, Ssemaganda A, Muwanga M, Wesonga I, Miiro G (2016). Low prevalence of laboratory-confirmed malaria in clinically diagnosed adult women from the Wakiso district of Uganda. Malar J.

[133] Barnabas RV, Webb EL, Weiss HA, Wasserheit JN (2011). The role of coinfections in HIV epidemic trajectory and positive prevention: a systematic review and meta-analysis. AIDS..

[134] Nielsen NO, Simonsen PE, Magnussen P, Magesa S, Friis H (2006). Cross-sectional relationship between HIV, lymphatic filariasis and other parasitic infections in adults in coastal northeastern Tanzania. Trans R Soc Trop Med Hyg.

[135] Cuadros DF, Branscum AJ, Crowley PH (2011). HIV-malaria co-infection: effects of malaria on the prevalence of HIV in East sub-Saharan Africa. Int J Epidemiol.

[136] Cuadros DF, Branscum AJ, Garcia-Ramos G (2011). No evidence of association between HIV-1 and malaria in populations with low HIV-1 prevalence. PLoS One.

[137] Cuadros DF, Garcia-Ramos G (2012). Variable effect of co-infection on the HIV infectivity: within-host dynamics and epidemiological significance. Theor Biol Med Model.

[138] Kublin JG, Patnaik P, Jere CS, Miller WC, Hoffman IF, Chimbiya N (2005). Effect of Plasmodium falciparum malaria on concentration of HIV-1-RNA in the blood of adults in rural Malawi: a prospective cohort study. Lancet..

[139] Abu-Raddad LJ, Patnaik P, Kublin JG (2006). Dual infection with HIV and malaria fuels the spread of both diseases in sub-Saharan Africa. Science..

[140] Xiao L, Owen SM, Rudolph DL, Lal RB, Lal AA (1998). Plasmodium falciparum antigen-induced human immunodeficiency virus type 1 replication is mediated through induction of tumor necrosis factor-alpha. J Infect Dis.

[141] Orlov M, Vaida F, Finney OC, Smith DM, Talley AK, Wang R (2012). P. falciparum enhances HIV replication in an experimental malaria challenge system. PLoS One.

[142] Hoffman IF, Jere CS, Taylor TE, Munthali P, Dyer JR, Wirima JJ (1999). The effect of Plasmodium falciparum malaria on HIV-1 RNA blood plasma concentration. AIDS..

[143] Wilairatana P, Meddings JB, Ho M, Vannaphan S, Looareesuwan S (1997). Increased gastrointestinal permeability in patients with Plasmodium falciparum malaria. Clin Infect Dis.

[144] Olupot-Olupot P, Urban BC, Jemutai J, Nteziyaremye J, Fanjo HM, Karanja H (2013). Endotoxaemia is common in children with Plasmodium falciparum malaria. BMC Infect Dis.

[145] Church JA, Nyamako L, Olupot-Olupot P, Maitland K, Urban BC (2016). Increased adhesion of Plasmodium falciparum infected erythrocytes to ICAM-1 in children with acute intestinal injury. Malar J.

[146] Potts RA, Tiffany CM, Pakpour N, Lokken KL, Tiffany CR, Cheung K (2016). Mast cells and histamine alter intestinal permeability during malaria parasite infection. Immunobiology..

[147] Chau JY, Tiffany CM, Nimishakavi S, Lawrence JA, Pakpour N, Mooney JP (2013). Malaria-associated L-arginine deficiency induces mast cell-associated disruption to intestinal barrier defenses against nontyphoidal Salmonella bacteremia. Infect Immun.

[148] Hotez PJ, Brindley PJ, Bethony JM, King CH, Pearce EJ, Jacobson J (2008). Helminth infections: the great neglected tropical diseases. J Clin Invest.

[149] Feldmeier H, Krantz I, Poggensee G (1994). Female genital schistosomiasis as a risk-factor for the transmission of HIV. Int J STD AIDS.

[150] Bentwich Z, Kalinkovich A, Weisman Z (1995). Immune activation is a dominant factor in the pathogenesis of African AIDS. Immunol Today.

[151] Kalinkovich A, Borkow G, Weisman Z, Tsimanis A, Stein M, Bentwich Z (2001). Increased CCR5 and CXCR4 expression in Ethiopians living in Israel: environmental and constitutive factors. Clin Immunol.

[152] Kalinkovich A, Weisman Z, Greenberg Z, Nahmias J, Eitan S, Stein M (1998). Decreased CD4 and increased CD8 counts with T cell activation is associated with chronic helminth infection. Clin Exp Immunol.

[153] Chachage M, Podola L, Clowes P, Nsojo A, Bauer A, Mgaya O (2014). Helminth-associated systemic immune activation and HIV co-receptor expression: response to albendazole/praziquantel treatment. PLoS Negl Trop Dis.

[154] Kroidl I, Chachage M, Mnkai J, Nsojo A, Berninghoff M, Verweij JJ (2019). Wuchereria bancrofti infection is linked to systemic activation of CD4 and CD8 T cells. PLoS Negl Trop Dis.

[155] Onguru D, Liang Y, Griffith Q, Nikolajczyk B, Mwinzi P, Ganley-Leal L (2011). Human schistosomiasis is associated with endotoxemia and Toll-like receptor 2- and 4-bearing B cells. Am J Trop Med Hyg.

[156] George PJ, Anuradha R, Kumar NP, Kumaraswami V, Nutman TB, Babu S (2012). Evidence of microbial translocation associated with perturbations in T cell and antigen-presenting cell homeostasis in hookworm infections. PLoS Negl Trop Dis.

[157] McDonald EA, Pond-Tor S, Jarilla B, Sagliba MJ, Gonzal A, Amoylen AJ (2014). Schistosomiasis japonica during pregnancy is associated with elevated endotoxin levels in maternal and placental compartments. J Infect Dis.

[158] Kurtis JD, Higashi A, Wu HW, Gundogan F, McDonald EA, Sharma S (2011). Maternal Schistosomiasis japonica is associated with maternal, placental, and fetal inflammation. Infect Immun.

[159] Coakley G, Buck AH, Maizels RM (2016). Host parasite communications-Messages from helminths for the immune system: Parasite communication and cell-cell interactions. Mol Biochem Parasitol.

[160] Mishra PK, Palma M, Bleich D, Loke P, Gause WC (2014). Systemic impact of intestinal helminth infections. Mucosal Immunol.

[161] Mwatha JK, Kimani G, Kamau T, Mbugua GG, Ouma JH, Mumo J (1998). High levels of TNF, soluble TNF receptors, soluble ICAM-1, and IFN-gamma, but low levels of IL-5, are associated with hepatosplenic disease in human schistosomiasis mansoni. J Immunol.

[162] Booth M, Mwatha JK, Joseph S, Jones FM, Kadzo H, Ireri E (2004). Periportal fibrosis in human Schistosoma mansoni infection is associated with low IL-10, low IFN-gamma, high TNF-alpha, or low RANTES, depending on age and gender. J Immunol.

[163] Salgame P, Yap GS, Gause WC (2013). Effect of helminth-induced immunity on infections with microbial pathogens. Nat Immunol.

[164] Clerici M, Shearer GM (1993). A TH1-->TH2 switch is a critical step in the etiology of HIV infection. Immunol Today.

[165] Olaitan A, Johnson MA, Reid WM, Poulter LW (1998). Changes to the cytokine microenvironment in the genital tract mucosa of HIV+ women. Clin Exp Immunol.

[166] Pearce EJ, Caspar P, Grzych JM, Lewis FA, Sher A (1991). Downregulation of Th1 cytokine production accompanies induction of Th2 responses by a parasitic helminth, Schistosoma mansoni. J Exp Med.

[167] Larkin BM, Smith PM, Ponichtera HE, Shainheit MG, Rutitzky LI, Stadecker MJ (2012). Induction and regulation of pathogenic Th17 cell responses in schistosomiasis. Semin Immunopathol.

[168] Mbow M, Larkin BM, Meurs L, Wammes LJ, de Jong SE, Labuda LA (2013). T-helper 17 cells are associated with pathology in human schistosomiasis. J Infect Dis.

[169] McFarlane AJ, McSorley HJ, Davidson DJ, Fitch PM, Errington C, Mackenzie KJ (2017). Enteric helminth-induced type I interferon signaling protects against pulmonary virus infection through interaction with the microbiota. J Allergy Clin Immunol.

[170] Scheer S, Krempl C, Kallfass C, Frey S, Jakob T, Mouahid G (2014). S. mansoni bolsters anti-viral immunity in the murine respiratory tract. PLoS One.

[171] Maizels RM, McSorley HJ (2016). Regulation of the host immune system by helminth parasites. J Allergy Clin Immunol.

[172] Ji MJ, Su C, Wu HW, Zhu X, Cai XP, Li CL (2003). Gene expression profile of CD4+ T cells reveals an interferon signaling suppression associated with progression of experimental Schistosoma japonicum infection. Cell Immunol.

[173] Sriram U, Xu J, Chain RW, Varghese L, Chakhtoura M, Bennett HL (2014). IL-4 suppresses the responses to TLR7 and TLR9 stimulation and increases the permissiveness to retroviral infection of murine conventional dendritic cells. PLoS One.

[174] Osborne LC, Monticelli LA, Nice TJ, Sutherland TE, Siracusa MC, Hepworth MR (2014). Coinfection. Virus-helminth coinfection reveals a microbiota-independent mechanism of immunomodulation. Science..

[175] Anahtar MN, Byrne EH, Doherty KE, Bowman BA, Yamamoto HS, Soumillon M (2015). Cervicovaginal bacteria are a major modulator of host inflammatory responses in the female genital tract. Immunity..

[176] WHO. Lymphatic filariasis Geneva, World Health Organization; [updated 2018; Accessed. Available from: http://www.who.int/lymphatic_filariasis/en/.

[177] Gopinath R, Ostrowski M, Justement SJ, Fauci AS, Nutman TB (2000). Filarial infections increase susceptibility to human immunodeficiency virus infection in peripheral blood mononuclear cells in vitro. J Infect Dis.

[178] Kroidl I, Saathof E, Maganga L, Clowes P, Maboko L, Hoerauf A (2016). Prevalence of Lymphatic Filariasis and Treatment Effectiveness of Albendazole/ Ivermectin in Individuals with HIV Co-infection in Southwest-Tanzania. PLoS Negl Trop Dis.

[179] Tafatatha T, Taegtmeyer M, Ngwira B, Phiri A, Kondowe M, Piston W (2015). Human Immunodeficiency Virus, Antiretroviral Therapy and Markers of Lymphatic Filariasis Infection: A Cross-sectional Study in Rural Northern Malawi. PLoS Negl Trop Dis.

[180] Kroidl I, Saathoff E, Maganga L, Makunde WH, Hoerauf A, Geldmacher C (2016). Effect of Wuchereria bancrofti infection on HIV incidence in southwest Tanzania: a prospective cohort study. Lancet..

[181] Colley DG, Andros TS, Campbell CH (2017). Schistosomiasis is more prevalent than previously thought: what does it mean for public health goals, policies, strategies, guidelines and intervention programs?. Infect Dis Poverty.

[182] Whitmee S, Haines A, Beyrer C, Boltz F, Capon AG, de Souza Dias BF (2015). Safeguarding human health in the Anthropocene epoch: report of The Rockefeller Foundation-Lancet Commission on planetary health. Lancet..

[183] Gryseels B, Polman K, Clerinx J, Kestens L (2006). Human schistosomiasis. Lancet..

[184] Allen JE, Wynn TA (2011). Evolution of Th2 immunity: a rapid repair response to tissue destructive pathogens. PLoS Pathog.

[185] Colley DG, Bustinduy AL, Secor WE, King CH (2014). Human schistosomiasis. Lancet..

[186] Turner JD, Jenkins GR, Hogg KG, Aynsley SA, Paveley RA, Cook PC (2011). CD4+CD25+ regulatory cells contribute to the regulation of colonic Th2 granulomatous pathology caused by schistosome infection. PLoS Negl Trop Dis.

[187] Anthony RM, Rutitzky LI, Urban JF, Stadecker MJ, Gause WC (2007). Protective immune mechanisms in helminth infection. Nat Rev Immunol.

[188] Downs JA, van Dam GJ, Changalucha JM, Corstjens PL, Peck RN, de Dood CJ (2012). Association of Schistosomiasis and HIV infection in Tanzania. Am J Trop Med Hyg.

[189] Mbabazi PS, Andan O, Fitzgerald DW, Chitsulo L, Engels D, Downs JA (2011). Examining the relationship between urogenital schistosomiasis and HIV infection. PLoS Negl Trop Dis.

[190] WHO. Female genital schistosomiasis: a pocket atlas for clinical health-care professionals Geneva, World Health Organization; [updated 2018; Accessed. Available from: http://apps.who.int/iris/handle/10665/180863.

[191] Mbah MLN, Poolman EM, Drain PK, Coffee MP, van der Werf MJ, Galvani AP (2013). HIV and Schistosoma haematobium prevalences correlate in sub-Saharan Africa. Tropical Med Int Health.

[192] Brodish PH, Singh K (2016). Association Between Schistosoma haematobium Exposure and Human Immunodeficiency Virus Infection Among Females in Mozambique. Am J Trop Med Hyg.

[193] Saathoff E, Olsen A, Magnussen P, Kvalsvig JD, Becker W, Appleton CC (2004). Patterns of Schistosoma haematobium infection, impact of praziquantel treatment and re-infection after treatment in a cohort of schoolchildren from rural KwaZulu-Natal/South Africa. BMC Infect Dis.

[194] Pillay P, Taylor M, Zulu SG, Gundersen SG, Verweij JJ, Hoekstra P (2014). Real-time polymerase chain reaction for detection of Schistosoma DNA in small-volume urine samples reflects focal distribution of urogenital Schistosomiasis in primary school girls in KwaZulu Natal, South Africa. Am J Trop Med Hyg.

[195] Hegertun IE, Sulheim Gundersen KM, Kleppa E, Zulu SG, Gundersen SG, Taylor M (2013). S. haematobium as a common cause of genital morbidity in girls: a cross-sectional study of children in South Africa. PLoS Negl Trop Dis.

[196] Adenowo AF, Oyinloye BE, Ogunyinka BI, Kappo AP (2015). Impact of human schistosomiasis in sub-Saharan Africa. Braz J Infect Dis.

[197] Ndhlovu PD, Mduluza T, Kjetland EF, Midzi N, Nyanga L, Gundersen SG (2007). Prevalence of urinary schistosomiasis and HIV in females living in a rural community of Zimbabwe: does age matter?. Trans R Soc Trop Med Hyg.

[198] Downs JA, Mguta C, Kaatano GM, Mitchell KB, Bang H, Simplice H (2011). Urogenital schistosomiasis in women of reproductive age in Tanzania's Lake Victoria region. Am J Trop Med Hyg.

[199] Wall KM, Kilembe W, Vwalika B, Dinh C, Livingston P, Lee YM (2018). Schistosomiasis is associated with incident HIV transmission and death in Zambia. PLoS Negl Trop Dis.

[200] Bochner AF (2018). Evaluating risk factors and synergistic effects of two common HIV-1 coinfections: schistosomiasis and trichomoniasis: University of Washington.

[201] Poggensee G, Kiwelu I, Weger V, Goppner D, Diedrich T, Krantz I (2000). Female genital schistosomiasis of the lower genital tract: prevalence and disease-associated morbidity in northern Tanzania. J Infect Dis.

[202] Kjetland EF, Ndhlovu PD, Mduluza T, Gomo E, Gwanzura L, Mason PR (2005). Simple clinical manifestations of genital Schistosoma haematobium infection in rural Zimbabwean women. Am J Trop Med Hyg.

[203] Kleppa E, Ramsuran V, Zulu S, Karlsen GH, Bere A, Passmore JA (2014). Effect of female genital schistosomiasis and anti-schistosomal treatment on monocytes, CD4+ T-cells and CCR5 expression in the female genital tract. PLoS One.

[204] Kleppa E, Klinge KF, Galaphaththi-Arachchige HN, Holmen SD, Lillebo K, Onsrud M (2015). Schistosoma haematobium infection and CD4+ T-cell levels: a cross-sectional study of young South African women. PLoS One.

[205] Dupnik KM, Reust MJ, Vick KM, Yao B, Miyaye D, Lyimo E (2019). Gene Expression Differences in Host Response to Schistosoma haematobium Infection. Infect Immun.

[206] Dupnik KM, Lee MH, Mishra P, Reust MJ, Colombe S, Haider SR, et al. Altered cervical mucosal gene expression and lower IL-15 levels in women with S. haematobium but not S. mansoni infection. J Infect Dis. 2018. 10.1093/infdis/jiy742 PMC6500550 30590736

[207] Mueller YM, Bojczuk PM, Halstead ES, Kim AH, Witek J, Altman JD (2003). IL-15 enhances survival and function of HIV-specific CD8+ T cells. Blood..

[208] Garrido C, Abad-Fernandez M, Tuyishime M, Pollara JJ, Ferrari G, Soriano-Sarabia N (2018). Interleukin-15-Stimulated Natural Killer Cells Clear HIV-1-Infected Cells following Latency Reversal Ex Vivo. J Virol.

[209] Manganaro L, Hong P, Hernandez MM, Argyle D, Mulder LCF, Potla U (2018). IL-15 regulates susceptibility of CD4(+) T cells to HIV infection. Proc Natl Acad Sci U S A.

[210] Leutscher PD, Pedersen M, Raharisolo C, Jensen JS, Hoffmann S, Lisse I (2005). Increased prevalence of leukocytes and elevated cytokine levels in semen from Schistosoma haematobium-infected individuals. J Infect Dis.

[211] Stecher CW, Kallestrup P, Kjetland EF, Vennervald B, Petersen E (2015). Considering treatment of male genital schistosomiasis as a tool for future HIV prevention: a systematic review. Int J Public Health.

[212] Agnew AM, Murare HM, Doenhoff MJ (1993). Immune attrition of adult schistosomes. Parasite Immunol.

[213] Cheever AW, Kamel IA, Elwi AM, Mosimann JE, Danner R, Sippel JE (1978). Schistosoma mansoni and S. haematobium infections in Egypt. III. Extrahepatic pathology. Am J Trop Med Hyg.

[214] Cheever AW (1968). A quantitative post-mortem study of Schistosomiasis mansoni in man. Am J Trop Med Hyg.

[215] Downs JA, Dupnik KM, van Dam GJ, Urassa M, Lutonja P, Kornelis D (2017). Effects of schistosomiasis on susceptibility to HIV-1 infection and HIV-1 viral load at HIV-1 seroconversion: A nested case-control study. PLoS Negl Trop Dis.

[216] Downs JA, de Dood CJ, Dee HE, McGeehan M, Khan H, Marenga A (2017). Schistosomiasis and Human Immunodeficiency Virus in Men in Tanzania. Am J Trop Med Hyg.

[217] Stabinski L, Reynolds SJ, Ocama P, Laeyendecker O, Ndyanabo A, Kiggundu V (2011). High prevalence of liver fibrosis associated with HIV infection: a study in rural Rakai. Uganda Antivir Ther.

[218] Sanya RE, Muhangi L, Nampijja M, Nannozi V, Nakawungu PK, Abayo E (2015). Schistosoma mansoni and HIV infection in a Ugandan population with high HIV and helminth prevalence. Tropical Med Int Health.

[219] Ssetaala A, Nakiyingi-Miiro J, Asiki G, Kyakuwa N, Mpendo J, Van Dam GJ (2015). Schistosoma mansoni and HIV acquisition in fishing communities of Lake Victoria, Uganda: a nested case-control study. Tropical Med Int Health.

[220] United Nations Department of Economic and Social Affairs, Population Division. World Contraceptive Use 2017; 2017 [Accessed 22 Feb 2018]. Available from: http://www.un.org/en/development/desa/population/publications/dataset/contraception/wcu2017.shtml.

[221] Dennis ML, Radovich E, Wong KLM, Owolabi O, Cavallaro FL, Mbizvo MT (2017). Pathways to increased coverage: an analysis of time trends in contraceptive need and use among adolescents and young women in Kenya, Rwanda, Tanzania, and Uganda. Reprod Health.

[222] Hall OJ, Klein SL (2017). Progesterone-based compounds affect immune responses and susceptibility to infections at diverse mucosal sites. Mucosal Immunol.

[223] Yegorov S, Galiwango RM, Good SV, Mpendo J, Tannich E, Boggild AK (2018). Schistosoma mansoni infection and socio-behavioural predictors of HIV risk: a cross-sectional study in women from Uganda. BMC Infect Dis.

[224] Siddappa NB, Hemashettar G, Shanmuganathan V, Semenya AA, Sweeney ED, Paul KS (2011). Schistosoma mansoni enhances host susceptibility to mucosal but not intravenous challenge by R5 Clade C SHIV. PLoS Negl Trop Dis.

[225] Chenine AL, Shai-Kobiler E, Steele LN, Ong H, Augostini P, Song R (2008). Acute Schistosoma mansoni infection increases susceptibility to systemic SHIV clade C infection in rhesus macaques after mucosal virus exposure. PLoS Negl Trop Dis.

[226] Yegorov S, Joag V, Galiwango RM, Good SV, Mpendo J, Tannich E (2019). Schistosoma mansoni treatment reduces HIV entry into cervical CD4+ T cells and induces IFN-I pathways. Nat Commun.

[227] Secor WE, Shah A, Mwinzi PM, Ndenga BA, Watta CO, Karanja DM (2003). Increased density of human immunodeficiency virus type 1 coreceptors CCR5 and CXCR4 on the surfaces of CD4(+) T cells and monocytes of patients with Schistosoma mansoni infection. Infect Immun.

[228] Prodger JL, Ssemaganda A, Ssetaala A, Kitandwe PK, Muyanja E, Mpendo J (2015). Schistosoma mansoni Infection in Ugandan Men Is Associated with Increased Abundance and Function of HIV Target Cells in Blood, but Not the Foreskin: A Cross-sectional Study. PLoS Negl Trop Dis.

[229] Berry A (1976). Multispecies schistosomal infections of the female genital tract detected in cytology smears. Acta Cytol.

[230] Gelfand M, Ross WF (1953). II. The distribution of schistosome ova in the genito-urinary tract in subjects of bilharziasis. Trans R Soc Trop Med Hyg.

[231] Feldmeier H, Daccal RC, Martins MJ, Soares V, Martins R (1998). Genital manifestations of schistosomiasis mansoni in women: important but neglected. Mem Inst Oswaldo Cruz.

[232] Smith JH, Elwi A, Kamel IA, von Lichtenberg F (1975). A quantitative post mortem analysis of urinary schistosomiasis in Egypt. II. Evolution and epidemiology. Am J Trop Med Hyg.

[233] Edington GM, Nwabuebo I, Junaid TA (1975). The pathology of schistosomiasis in Ibadan, Nigeria with special reference to the appendix, brain, pancreas and genital organs. Trans R Soc Trop Med Hyg.

[234] Colombe S, Lee MH, Masikini PJ, van Lieshout L, de Dood CJ, Hoekstra PT, et al. Decreased Sensitivity of Schistosoma sp. Egg Microscopy in Women and HIV-Infected Individuals. Am J Trop Med Hyg. 2018. 10.4269/ajtmh.17-0790 PMC5928829 29405114

[235] Yepes E, Varela MR, Lopez-Aban J, Rojas-Caraballo J, Muro A, Mollinedo F (2015). Inhibition of Granulomatous Inflammation and Prophylactic Treatment of Schistosomiasis with a Combination of Edelfosine and Praziquantel. PLoS Negl Trop Dis.

[236] Schmiedel Y, Mombo-Ngoma G, Labuda LA, Janse JJ, de Gier B, Adegnika AA (2015). CD4+CD25hiFOXP3+ Regulatory T Cells and Cytokine Responses in Human Schistosomiasis before and after Treatment with Praziquantel. PLoS Negl Trop Dis.

[237] Pattacini L, Baeten JM, Thomas KK, Fluharty TR, Murnane PM, Donnell D (2016). Regulatory T-Cell Activity But Not Conventional HIV-Specific T-Cell Responses Are Associated With Protection From HIV-1 Infection. J Acquir Immune Defic Syndr.

[238] Ndeffo Mbah ML, Poolman EM, Atkins KE, Orenstein EW, Meyers LA, Townsend JP (2013). Potential cost-effectiveness of schistosomiasis treatment for reducing HIV transmission in Africa--the case of Zimbabwean women. PLoS Negl Trop Dis.

[239] Ndeffo Mbah ML, Gilbert JA, Galvani AP (2014). Evaluating the potential impact of mass praziquantel administration for HIV prevention in Schistosoma haematobium high-risk communities. Epidemics..

[240] Joag VR, McKinnon LR, Liu J, Kidane ST, Yudin MH, Nyanga B, et al. Identification of preferential CD4 T-cell targets for HIV infection in the cervix. Mucosal Immunol. 2015. 10.1038/mi.2015.28 25872482

[241] Veazey RS, Mansfield KG, Tham IC, Carville AC, Shvetz DE, Forand AE (2000). Dynamics of CCR5 expression by CD4(+) T cells in lymphoid tissues during simian immunodeficiency virus infection. J Virol.

[242] Sandler NG, Bosinger SE, Estes JD, Zhu RT, Tharp GK, Boritz E (2014). Type I interferon responses in rhesus macaques prevent SIV infection and slow disease progression. Nature..

[243] Foster TL, Pickering S, Neil SJD (2017). Inhibiting the Ins and Outs of HIV Replication: Cell-Intrinsic Antiretroviral Restrictions at the Plasma Membrane. Front Immunol.

[244] Downs JA, Kabangila R, Verweij JJ, Jaka H, Peck RN, Kalluvya SE (2013). Detectable urogenital schistosome DNA and cervical abnormalities 6 months after single-dose praziquantel in women with Schistosoma haematobium infection. Tropical Med Int Health.

